# Capturing irreversible photoswitching in *trans*-cinnamic acid by small molecule synchrotron serial crystallography

**DOI:** 10.1039/d6cc02988d

**Published:** 2026-08-11

**Authors:** Sam G. Lewis, Ben A. Coulson, Debashish Das, Gabriel Karras, Mark R. Warren, Lauren E. Hatcher

**Affiliations:** a School of Chemistry, Cardiff University Main Building Park Place CF10 3AT UK HatcherL1@cardiff.ac.uk; b Diamond Light Source, Harwell Science and Innovation Campus Didcot Oxfordshire OX11 0DE UK

## Abstract

Small molecule synchrotron serial crystallography (smSSX) is used to monitor irreversible photodimerization in α-*trans*-cinnamic acid, enabling structure determination of intermediate states and quantification of batch-level reaction heterogeneity.

## Graphical abstract

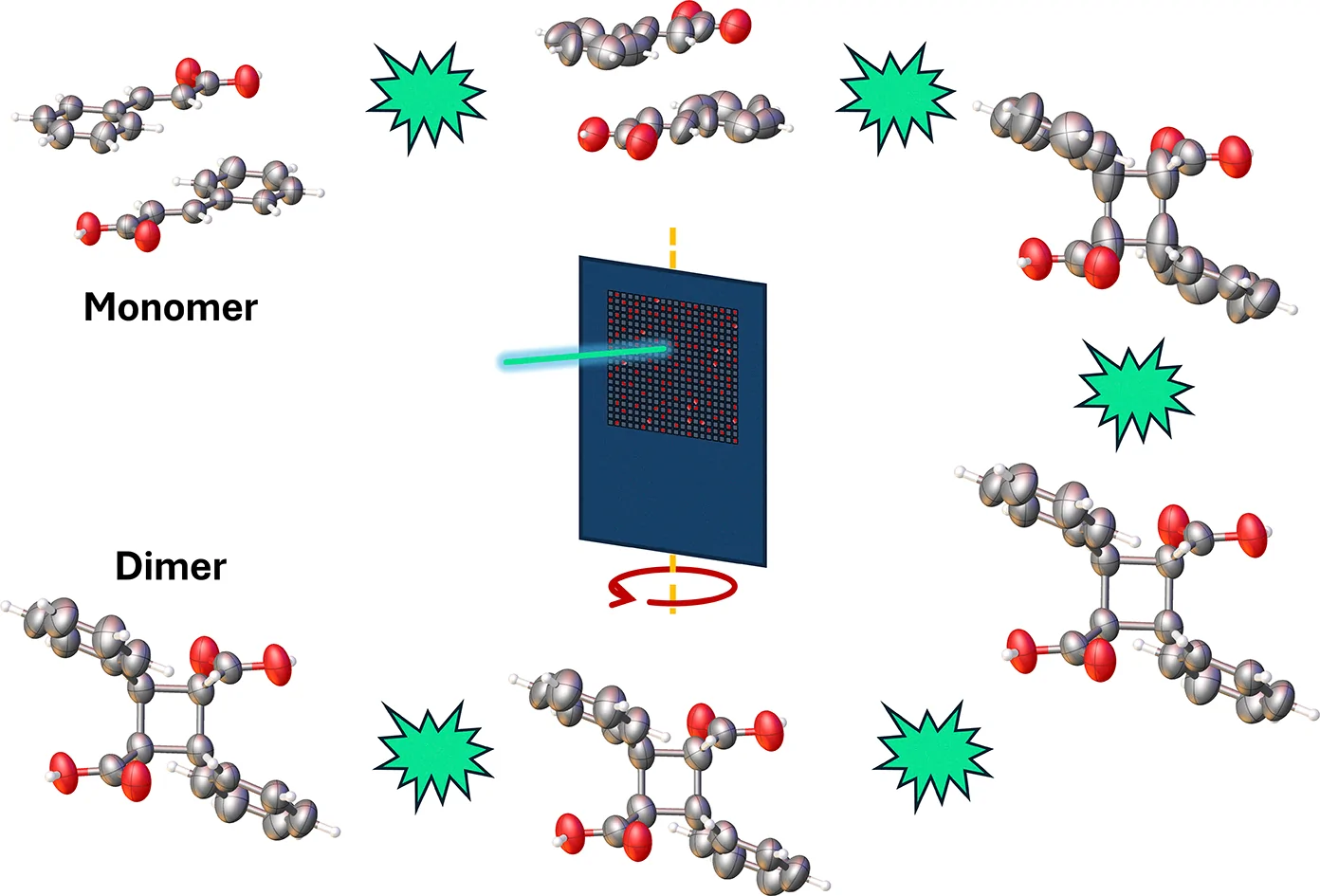

Photoswitchable molecular solids undergoing structural change in response to light are of significant interest for applications in data storage,^1,2^ molecular machines,^3–6^ smart coatings,^7,8^ sensors,^9–11^ electronics^12–14^ and solar energy.^15,16^ While many technologies rely on reversible switching between two distinct states, irreversible switches are equally valuable in contexts such as controlled drug delivery^17,18^ and safety devices, *e.g.* single-use sensors.^19,20^ Tracking reaction progress in 3D and at the atomic scale is vital to elucidate mechanisms and establish the structure–property relationships that underpin advanced materials design. Such insight is directly provided by *in situ* structural analysis methods, notably by photocrystallography.

Photocrystallographic experiments integrate a light source into X-ray diffraction equipment to enable *in situ* monitoring of light-induced structure changes in crystals.^21^ For rapid and reversible processes, stroboscopic pump–probe methods have been developed since the 1990s, initially at synchrotron facilities to study photoactive proteins.^22,23^ The first application to a small molecule crystal was reported by Coppens in 2002, revealing the structural origins of long-lived triplet phosphorescence in [Pt_2_(POP)_4_]^4−^.^24^ Subsequent studies have captured transient photoexcited species with lifetimes as short as picoseconds.^25,26^ However, these methods face persistent challenges, including crystal damage from repeated intense pump and probe pulses, low excited state populations and the inherent inability to study irreversible transformations *via* the stroboscopic technique. Time resolution in synchrotron pump–probe experiments is also typically limited to the picosecond regime by the intrinsic pulse structure of the source.

The advent of X-ray free-electron lasers (XFELs) in the early 2010s enables femtosecond resolution, allowing observation of the ultrafast structural dynamics following photoexcitation. However, the extreme peak energies of XFELs can destroy crystals in a single exposure, making stroboscopic methods impractical. Serial crystallography (SX) has emerged as a solution, employing a diffract-before-destroy approach where a single diffraction image is collected on each crystal and images from thousands of crystals are merged to deliver complete data for structure solution. First developed for proteins,^27–29^ SX is now being adapted for small molecules at XFEL^30–34^ and synchrotron sources.^35,36^ Progress has been hindered by sparse indexing challenges arising from the limited number of Bragg reflections recorded in a single diffraction image from a small molecule crystal. The lack of data per image complicates determination of unit cell parameters and crystal orientations, ultimately hindering data merging.^37,38^ In 2024, we introduced a strategy that circumvents sparse indexing for small molecule synchrotron serial crystallography (smSSX) by employing a fixed-target approach implementing small angular rotations at each grid position.^39^ This produces partial datasets consisting of a short, fine-sliced image series for each crystal, overcoming the reflection partiality problems inherent to single-shot SX ‘still’ images.^40^ Each partial dataset is reliably indexed *via* established small molecule routines to deliver accurate peak positions and precise unit cell parameters. Benchmarking of our small rotative fixed-target SSX (SR-FT-SSX) method on the inorganic photoswitch sodium nitroprusside delivered data to *d*_min_ < 0.6 Å, allowing *ab initio* structure solution with no known data input. Our tests indicate rotations as small as 0.3° (3 × 0.1°-wide images) are adequate for indexing, highlighting the potential of SR-FT-SSX for very photo or X-ray-sensitive samples.

In this article, we apply our SR-FT-SSX approach to a photocrystallographic study of irreversible photoswitching in α-*trans*-cinnamic acid (α-TCA). This polymorph, the subject of seminal *in situ* crystallographic studies by Schmidt and Cohen in the 1960s,^41–43^ undergoes single crystal [2+2] photodimerization to form a cyclobutane ring between neighbouring molecules, yielding the α stereoisomer of truxillic acid (Fig. 1a). While the ground state (dark) and final dimer structures are well documented,^44–51^ fewer intermediate structures are reported^46,50^ and reaction progress is more often monitored *via* changes in the unit cell parameters, notably the *β* angle.^49^ Here, we demonstrate that SSX delivers 3D structure characterisation at multiple intermediate stages of the transformation, as well as enabling a detailed statistical analysis of photoexcited state populations across a microcrystal batch. This batch-level insight, unique to SX, provides unprecedented understanding of photoreaction heterogeneity, with broad implications for the design and optimisation of photoactive crystals for real-world applications.

**Fig. 1:**
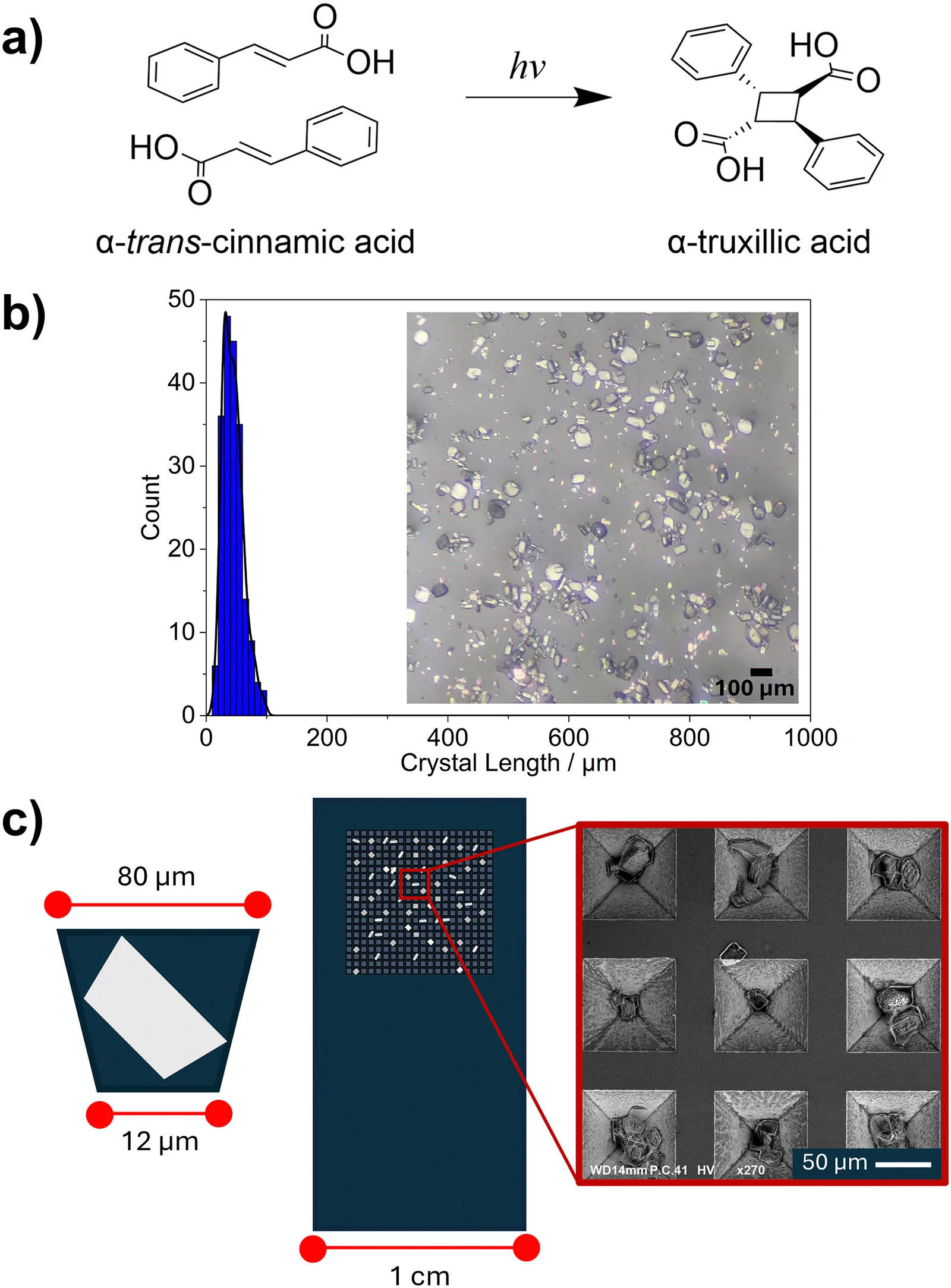
α-TCA sample preparation for smSSX. (a) [2+2] photodimerization of α-TCA: “head-to-tail” packing in the α-polymorph delivers the centrosymmetric stereoisomer α-truxillic acid as the photoproduct. (b) Optical photomicrograph of α-TCA crystals produced by antisolvent crash, with a narrow length distribution determined by digital image analysis. (c) Microcrystal loading in FT grids containing a 20 × 20 city block of 400 wells.

α-TCA microcrystals were prepared by antisolvent crash crystallization. A 25 wt% (0.25 g g^−1^) solution of TCA in acetone was rapidly injected into an excess of petroleum ether (40 : 60) under continuous ultrasonic agitation (see SI, Section S1). This yielded square plate-like microcrystals of narrow size distribution, with a median crystal length of 42 µm and median crystal width of 26 µm recorded across a representative batch (Fig. 1b and Fig. S1). *Ex situ* PXRD and FT-IR analyses confirm exclusive formation of the α-polymorph (Fig. S2). Microcrystals were suspended in 1 wt% sodium dodecyl sulfate in deionised water and a 1 µL aliquot was applied to the FT grid, gently spread to disperse the crystals and a light vacuum applied to remove excess solution. Although target crystal dimensions were selected to match the grid architecture (Fig. 1c), SEM imaging showed that multiple α-TCA crystals frequently occupied individual grid wells. Although suboptimal for serial photocrystallography, as overlapping crystals may attenuate the laser, this issue was manageable within the data processing workflow.

smSSX measurements were performed on Beamline I19 at Diamond Light Source. The ground state (dark) α-TCA structure was first collected using a two-step protocol (Fig. S3) adapted from our published SR-FT-SSX method.^39^ In step one, the FT grid was screened by rotating each well through a diffraction angle, *φ* = 1°, and recording 5 × 0.2° *φ*-scan images at all 400 positions using an attenuated X-ray beam. These SR partial datasets were processed on-the-fly *via* a semi-automated script that submits each dataset to DIALS^52^ for Bragg spot finding and outputs reflection counts per well. Wells recording >10 reflections were then selected for full SR-FT-SSX data collection. In step two, wider-angle datasets were recorded at the selected wells by rotating through *φ* = 60° and collecting 300 × 0.2° *φ*-scan images at higher X-ray transmission, aiming to maximise the diffraction intensity. This two-step method significantly reduces dead-time relative to our original SR-FT-SSX implementation by avoiding full collections on empty wells or those containing low-quality crystals. Full data collection information is given in the SI, Section S2.

For photocrystallographic data collections, *in situ* irradiation of the crystals in the grid was achieved using the PORTO femtosecond pulsed laser system.^53^ Prior work has shown that α-TCA photodimerization is readily achieved by two-photon excitation using green laser light, with the non-linear process enhancing homogeneous photoproduct formation throughout the crystal bulk.^49^ As such, this study used PORTO's second harmonic (*λ* = 515 nm) for two-photon excitation of α-TCA in the FT grids. The laser was operated at 50 kHz using 20% of the available pump power (3 mW) corresponding to a pulse energy of 0.06 µJ at the sample. Each selected well position was irradiated immediately prior to recording the corresponding SR partial dataset. Laser exposure was controlled by opening a manual shutter for a defined period, delivering a known number of laser pulses to each well. Full detail of the PORTO laser parameters is provided in the SI, Section S2. The laser beam was focussed to 80 × 70 µm (FWHM), closely matched to the 80 × 80 µm openings on the FT grid (Fig. 2). The 100 µm well-to-well spacing ensured exclusive irradiation of the target well, without contamination of adjacent positions. Photocrystallographic data collections were repeated at regular intervals, resulting in a series of defined laser exposure conditions (Table 1).

**Fig. 2:**
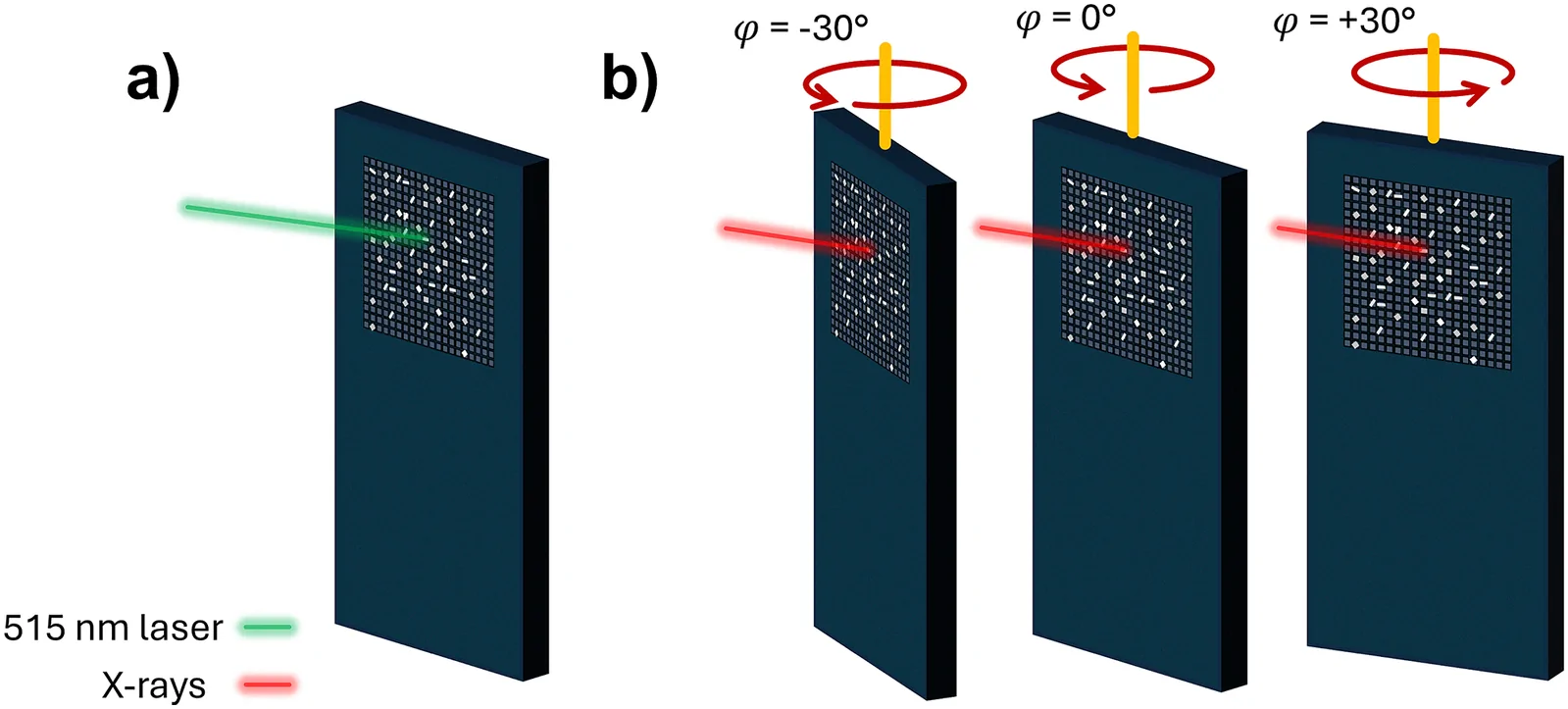
Schematic diagrams of *in situ* irradiation in the FT grid. (a) Irradiation of a well position: the laser is focussed on the grid face opposite the X-ray apertures, utilising the 80 × 80 µm windows. (b) Selected grid orientations across a 60° *φ*-scan.

**Table 1:** Summary of laser exposure conditions. PORTO pulse duration: 300 fs, repetition rate: 50 kHz. # laser pulses estimated as: [shutter open time] × [repetition rate]. Effective laser irradiation time estimated as: [pulse duration] × [total number of pulses delivered]

Condition #	Shutter open time [s]	# of laser pulses	Effective laser irradiation time [ns]
1	0	0	0
2	0.5	25 000	7.5
3	2	100 000	30
4	7	350 000	105
5	10	500 000	150
6	20	1000 000	300
7	100	5000 000	1500
8	200	10 000 000	3000

Bulk processing of the SR partial datasets was carried out using a modified version of our semi-automated SR-FT-SSX pipeline.^39^ This updated workflow incorporates multi-lattice indexing (Fig. S4),^54^ enabling reliable processing of the data from wells occupied by more than one crystal. Full details are provided in SI Section S3. The progress of the [2+2] cycloaddition was assessed by monitoring changes in unit cell parameters indexed from the data recorded under each laser exposure condition (1–8). The spread of *β* angles, the most diagnostic parameter, across the selected wells are visualised as box-and-whisker plots in Fig. 3a. As expected from prior work,^49^ the *β* angle increases with laser exposure and can be used as an indirect measure of photoreaction progress. Interestingly, the distribution of *β* angles across all crystals analysed shows considerable variation as a function of laser exposure. While condition 1 has an expectedly narrow *β* angle distribution for the dark-state monomer, irradiation induces an immediate broadening, reaching a maximum under condition 3 (100 000 laser pulses; interquartile range 1.560°, median *β* = 97.980°). Continued irradiation narrows the distribution once more, with the spread of values becoming near-negligible for conditions 7–8 as the reaction approaches full conversion to α-truxillic acid. Full detail of the statistical analysis is provided in the SI (Table S2).

**Fig. 3:**
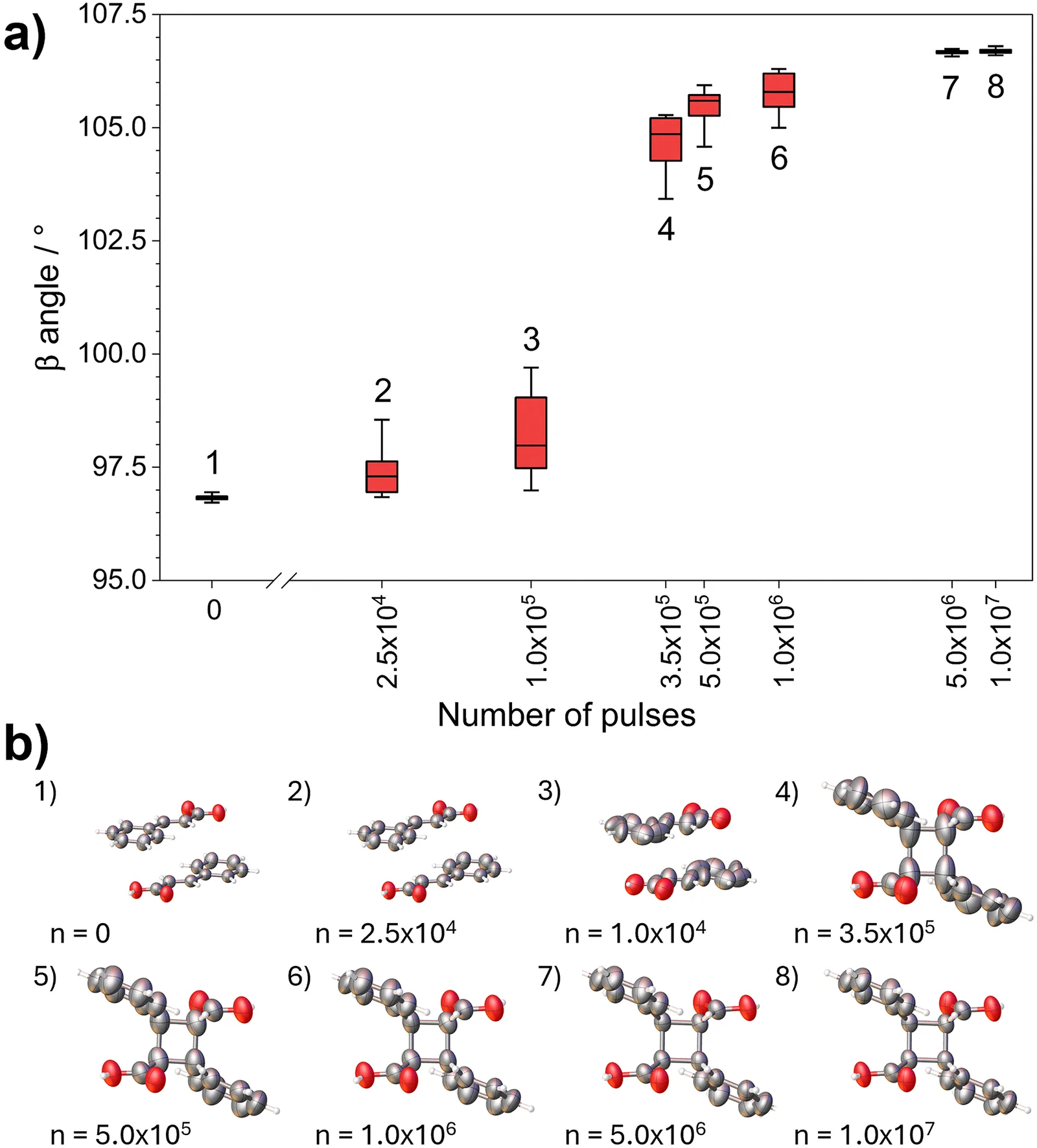
Results of *in situ* smSSX photocrystallography with α-TCA. (a) Box-and-whisker plots showing the spread of *β* angles across the microcrystal batch (outliers omitted for clarity, line shows median *β* angle). Median *β* angles and dataset counts for 1–8: 96.817° (88), 97.299° (33), 97.980° (18), 104.860° (9), 105.595° (16), 105.970° (6), 106.673° (48), 106.690° (45). (b) Average X-ray crystal structures solved from final merged smSSX datasets for each of the 8 conditions, with ellipsoids shown at 50% probability. *n* = # of accumulated laser pulses.

These data provide a clear indication of a significant variation in photoresponse across the microcrystal batch. Individual crystals display markedly different magnitudes of *β* angle change under identical laser exposure, indicating substantial heterogeneity of photoreactivity from crystal to crystal. The *β* value spread seen likely reflects differences in intrinsic crystal attributes, such as size and shape, which affect surface-area-to-volume ratio and therefore laser penetration efficiency.^55^ Extrinsic factors, such as the orientation of each crystal within the well and the presence of multiple crystals at some positions, may further contribute to the variability. Such batch-level behaviour is critically important for real-world photoswitch applications, where materials are more typically deployed in micro- or poly-crystalline, rather than single-crystal form.^56,57^ Importantly, this population-wide insight is unique to our SSX approach; achieving comparable information with conventional single-crystal photocrystallography would require prohibitively time-consuming repeat experiments.

Processed partial datasets from conditions 1–8 were filtered to identify those suitable for scaling and merging into final serial X-ray datasets. Our previous SR-FT-SSX workflow used diffraction quality metrics such as the diffraction signal-to-noise [*I*/*σ*(*I*)] and residual (*R*) factors, as filtering criteria.^39^ However, applying this approach to the α-TCA datasets proved ineffective due to the substantial unit cell changes that arise from photodimerization. To address this, we adopted a strategy inspired by protein SX and employed the program *cosym* within the DIALS software suite for partial dataset filtering.^58^ This algorithm performed hierarchical clustering of the unit cell parameters from the indexed partial datasets to identify consistent subsets for each irradiation condition, which were then used as the basis for dataset filtering (see SI Section S3). Datasets were retained if (i) unit cell parameters were within ±0.5 Å or ° of the median values for the condition and (ii) *I*/*σ*(*I*) ≥ 2 to exclude weak diffraction data, with thresholds informed by initial testing (sensitivity analysis in SI Section S5). The resulting subsets were scaled and merged in DIALS to generate final combined datasets for 1–8, balancing data quality with completeness. Structures were solved and refined using SHELXT^59^ and SHELXL.^60^ Structure solution and refinement details are given in SI Section S4.

Several limitations of the merged refinements should be noted. The maximum diffraction resolution decreased from 0.8 Å in condition 1 to nearer 1.2 Å for intermediate states (conditions 2–6), consistent with the increased structural disorder resulting from partial conversion of the monomeric ground state into the cyclobutane photodimer. Resolution returned to 0.8 Å once full photoconversion was achieved, indicating that the temporary reduction in diffraction power arises from transient disorder in the mixed TCA/truxillic acid state. Additionally, fewer partial datasets were identified as suitable for merging in conditions 2–6, reducing the total number of observations in the merged datasets and weakening the data-to-parameter ratio for the intermediate structure models. To avoid over-interpretation, no explicit disorder modelling was attempted for these conditions; instead, the carbon atoms of the C=C backbone (C(2), C(3)) were freely refined to represent the average electron density in each case. Table S4 lists full refinement metrics for each condition. The resulting serial crystal structures (Fig. 3b and Fig. S14–S21) are assembled into a “molecular movie” depicting the evolution of the average crystal structure with laser exposure (ESI).

In summary, this study demonstrates that smSSX is a robust and versatile approach for tracking irreversible solid-state reactions. The method is highly effective for probing variations in photoreactivity across a microcrystal batch, a capability that is especially important for studying functional microcrystalline materials, for which device performance may depend on the homogeneity of the photochemical response.^3^ smSSX provides statistically robust, batch-level insight into ensemble photoreactivity, while still delivering the 3D structural information traditional to single-crystal methods, making it especially powerful for intrinsically heterogeneous systems.

Although our combined structure models provide qualitative insight into average structural changes induced by irradiation, the data quality in this proof-of-concept study was insufficient to derive full disorder models to quantify batch-level population dynamics. Nevertheless, our results establish smSSX as a viable approach for batch solid-state photoreactivity analysis. Ongoing gains in X-ray flux, dataset-filtering and refinement will continue to enhance smSSX photocrystallography, expanding its scope toward increasingly complex photoreactions in the near future.

## Author contributions

LEH and MRW are responsible for conceptualization, supervision, funding acquisition and writing – review/editing. SGL is responsible for investigation, formal analysis, data curation and writing – original draft. GK, BC and DD carried out investigation and methodology.

## Conflicts of interest

There are no conflicts to declare.

## Supplementary Material

CC-062-D6CC02988D-s001

CC-062-D6CC02988D-s002

CC-062-D6CC02988D-s003

## Data Availability

Data processing code is available on GitHub *via*https://github.com/SGLXRD/Hatcher_Group/tree/main. All other raw data are provided *via* the Cardiff University Research Data Repository *via*https://doi.org/10.17035/cardiff.33225816. Supplementary information (SI) including crystallization information, crystal size distribution analysis, processed FTIR spectra and PXRD patterns and additional data collection and data processing information. See DOI: https://doi.org/10.1039/d6cc02988d. CCDC 2554295–2554302 contain the supplementary crystallographic data for this paper.^61*a*–*h*^
