# Supplementary material for: Capturing irreversible photoswitching in trans-cinnamic acid by small molecule synchrotron serial crystallography
Source: Chem Commun (Camb). 2026 Aug 11;62(71):17629–33. doi: 10.1039/d6cc02988d (PMC13564980; doi:10.1039/d6cc02988d)
Supplement: Supplementary file 1 [file CC-062-D6CC02988D-s001.pdf]

This journal is © The Royal Society of Chemistry 2026

Capturing Irreversible Photoswitching in *trans*-Cinnamic Acid by Small Molecule Synchrotron Serial Crystallography

Supplementary Information

Sam G. Lewis,[a,b] Ben A. Coulson,[b] Debashish Das,[a] Gabriel Karras,[b] Mark R. Warren*[b] and Lauren E. Hatcher*

[a] School of Chemistry, Cardiff University, Main Building, Park Place, CF10 3AT, United Kingdom. *HatcherL1@cardiff.ac.uk.
[b] Diamond Light Source, Harwell Science and Innovation Campus, Didcot Oxfordshire, OX11 0DE, United Kingdom.

Section 1: Batch microcrystallization of α-*trans*-cinnamic acid

Crystallization protocol

Microcrystalline α-*trans*-cinnamic acid (α-TCA) was prepared via rapid reverse antisolvent crash crystallisation, following a protocol analogous to that reported previously by us for sodium nitroprusside dihydrate.[1] A 25 wt% (0.25 g/g) stock solution of TCA (6.67 mmol) was prepared by dissolving 0.5934 g of TCA in acetone (5000 µL). Light petroleum (2000 µL) was placed in a septum-capped vial and equilibrated in an ultrasonic bath maintained at 20 °C. Under continuous sonication, 100 µL of the TCA–acetone solution was rapidly injected into the antisolvent using a syringe needle, inducing immediate crystallisation. Sonication was continued for an additional 30 s before cessation, after which the suspension was examined by optical microscopy for crystal size analysis (detailed below). The microcrystals were isolated by transferring 1000 µL of the suspension into two 1.5 mL microcentrifuge tubes and allowing the solids to settle for 5 min. The supernatant was carefully removed, and residual solvent was eliminated by gentle application of compressed air, affording 50 mg of dry microcrystalline product. The resulting material was used for ex situ phase purity assessment by FT-IR spectroscopy and powder X-ray diffraction (PXRD).

SSX sample preparation and delivery

The dried TCA microcrystals obtained as described above were prepared in advance of beamtime, transported in the dry state, and used for experiments on Beamline I19 at Diamond Light Source (DLS). Immediately prior to loading, 50 mg of microcrystals were resuspended in 500 µL of a 1 wt% aqueous sodium dodecyl sulfate (SDS) solution. An aliquot (1 µL) of this suspension was deposited onto the centre of a 400-well silicon fixed-target grid (20 × 20 array silicon nitride coated grids; purchased from the Southampton Nanofabrication Facility[2]) and evenly distributed across the surface using a pipette tip. Excess liquid was removed by applying a gentle vacuum from beneath the grid.

This resuspension protocol contrasts with our previous work on sodium nitroprusside dihydrate, in which microcrystals were suspended directly into a 1-butanol antisolvent immediately following crystallisation.[3] While TCA is soluble in most organic solvents and reported to exhibit negligible solubility in deionised water,[4] preliminary studies showed that extended suspension time in aqueous media led to observable crystal degradation. Consequently, resuspension was performed only immediately prior to sample delivery.

Despite these concerns, deionised water still provided the lowest solubility for TCA among the solvents evaluated thus was chosen as the most suitable solvent for resuspension. However, the microcrystals exhibited a strong tendency to agglomerate in pure aqueous suspension, limiting effective dispersion. To mitigate this, a detergent-assisted approach was adopted, inspired by the work of Brewster, Hohman, and co-workers, who demonstrated enhanced dispersion of n-alkanethiolate microcrystals in aqueous media upon addition of small amounts of surfactant.[5] Incorporation of 1 wt% sodium dodecyl sulfate (SDS) in deionised water was found to substantially improve dispersion of the TCA microcrystals.

Crystal size analysis by digital image processing

Crystal size analysis was performed using optical photomicrographs of the TCA microcrystals. Images were acquired using a commercially-available smartphone camera mounted on a Motic SMZ-161 series stereoscopic microscope. Measurements of crystal dimensions were carried out using the software ImageJ,[6] with crystal length and width determined as the orthogonal distances between opposing crystal faces. The larger of the two measured dimensions was assigned as the crystal length, while the smaller was taken as the width. Image calibration was achieved using a physical graticule.

Table S1 Crystal size distribution statistics for TCA microcrystals obtained by rapid reverse antisolvent crystallisation under continuous sonication using an acetone/light petroleum solvent/antisolvent system (initial TCA concentration in acetone: 25 wt% [0.25 g/g]). (a) Length, (b) width, and (c) aspect ratio.

Number of crystals measured	Metric	Mean value	Standard deviation	Median value
200	Aspect ratio	2.03	0.26	1.48
	Length / μm	44	16	42
	Width / μm	30	12	26

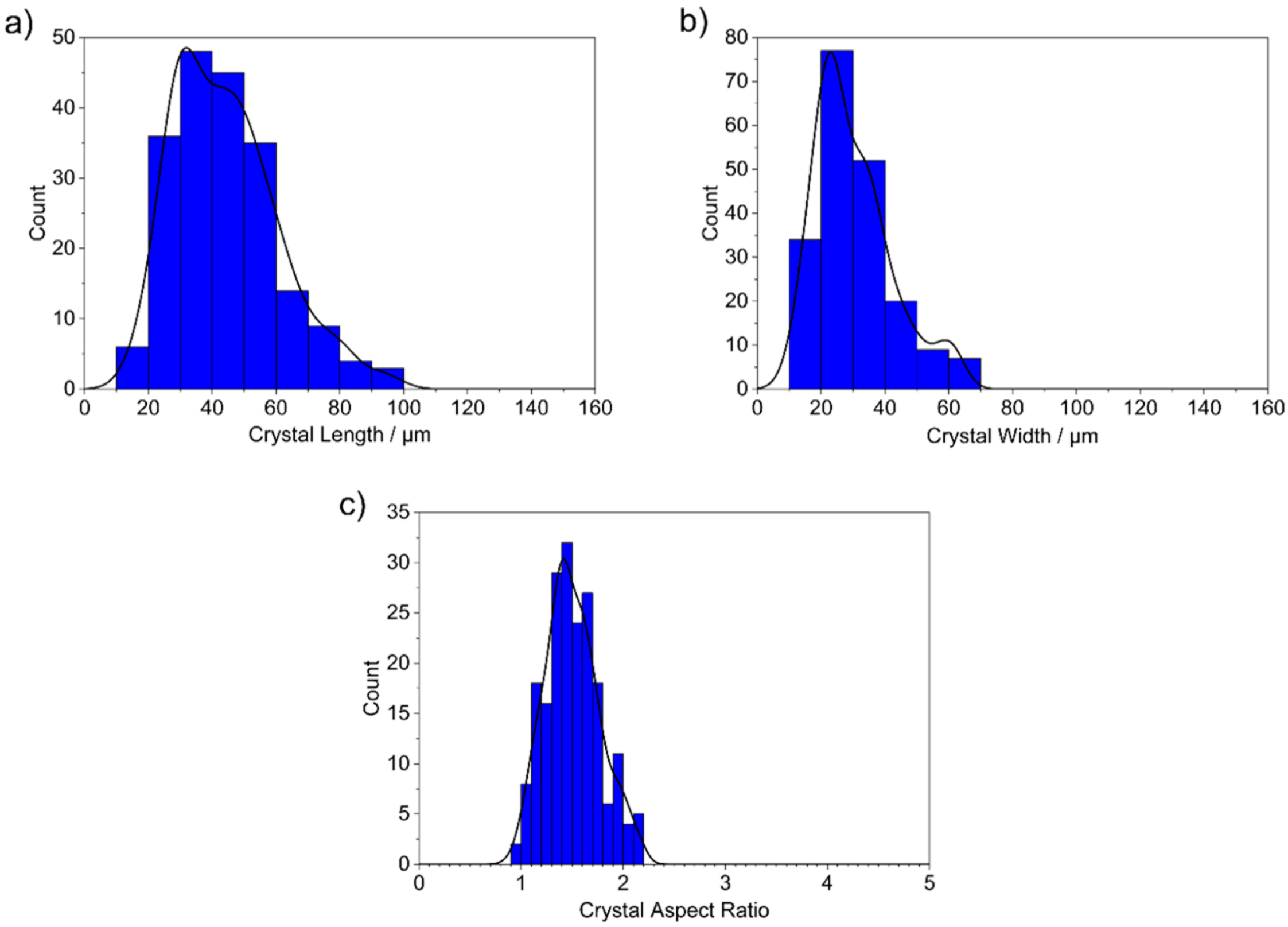

Figure S1 Crystal size distribution histograms for TCA microcrystals prepared by rapid reverse antisolvent crystallisation under continuous sonication from an acetone/light petroleum solvent/antisolvent system (initial TCA concentration in acetone: 25 wt% [0.25 g/g]). (a) Lengths, (b) widths, and (c) aspect ratios.

Ex-situ analysis of microcrystal batches

Instrument details:

FT-IR measurements were performed on a Shimadzu IRAffinity-1S FT-IR Spectrophotometer. Data were collected in % transmittance mode using Happ-Genzel apodization scanning over the range of 500–4000 cm^{-1} and the data averaged over 32 scans.

Powder X-ray diffraction (PXRD) data collections were conducted on a Rigaku XtaLab Synergy-R rotating anode diffractometer equipped with a CuKα (λ = 1.54056 Å, mirror monochromated) source scanning over a 2θ range of 10° to 40°. Data were recorded at ambient temperature (20 °C), with a small amount of powder (ca. 5 mg) placed onto a Kapton loop with a minimal amount of Fomblin oil.

Optical microscopy was performed using a Motic SMZ-161 Series microscope. A 1 mm graticule with 0.01mm graduations was used to provide scale.

Analysis:

Ex-situ characterisation was performed on dried microcrystal batches prepared as described above to confirm polymorph identity and verify that no phase transformation occurred during crystallization. FT-IR spectroscopy (Figure S2a) showed good agreement with literature spectra reported for α-TCA.[7] However, powder X-ray diffraction provides the most definitive means of distinguishing between the α and β polymorphs. As shown in Figure S2b, the experimental PXRD pattern closely matches that simulated from the reported single-crystal X-ray structure of α-TCA, with no significant peaks attributable to the β polymorph. Together, the FT-IR and PXRD data confirm formation of the α-polymorph.

a)

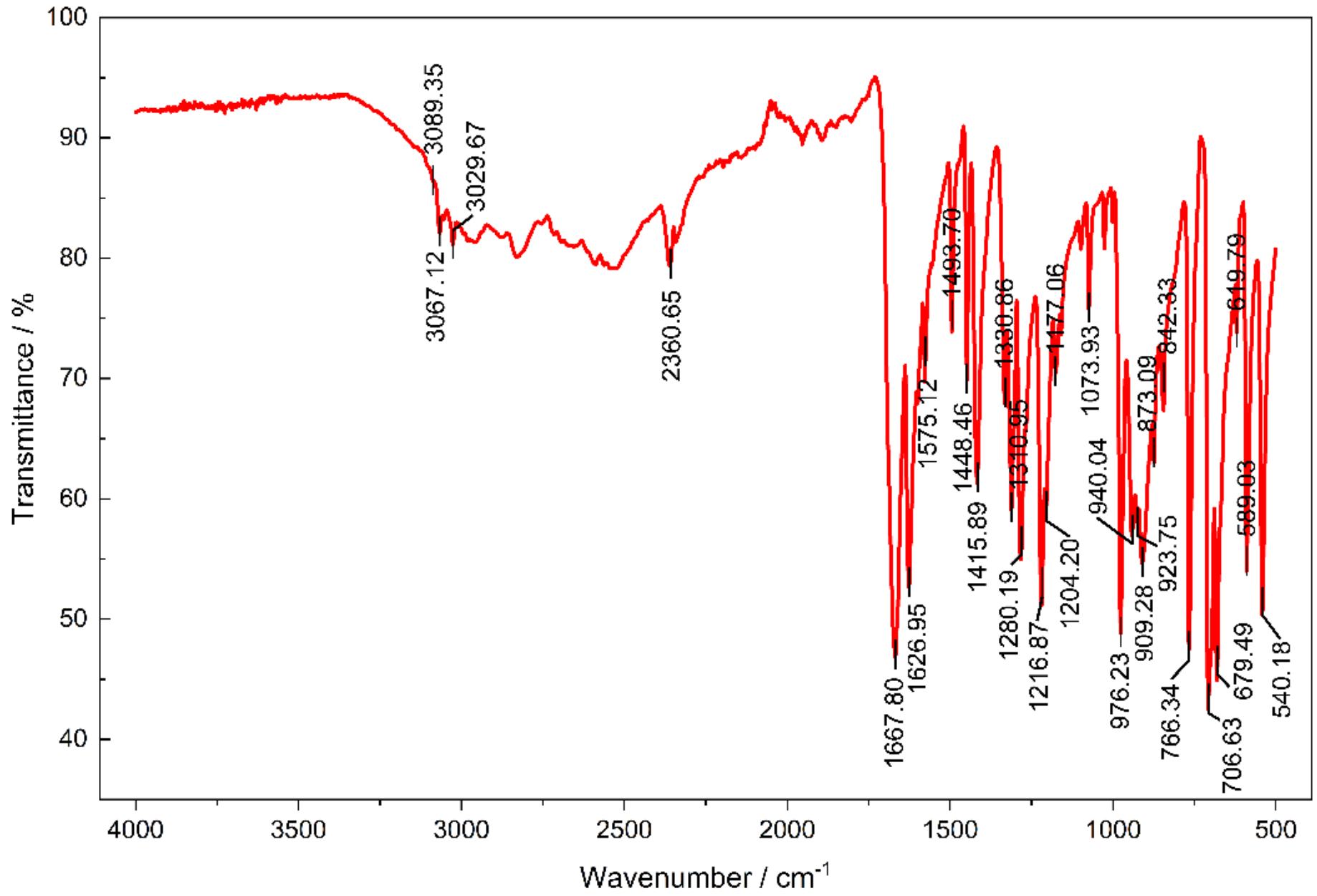

b)

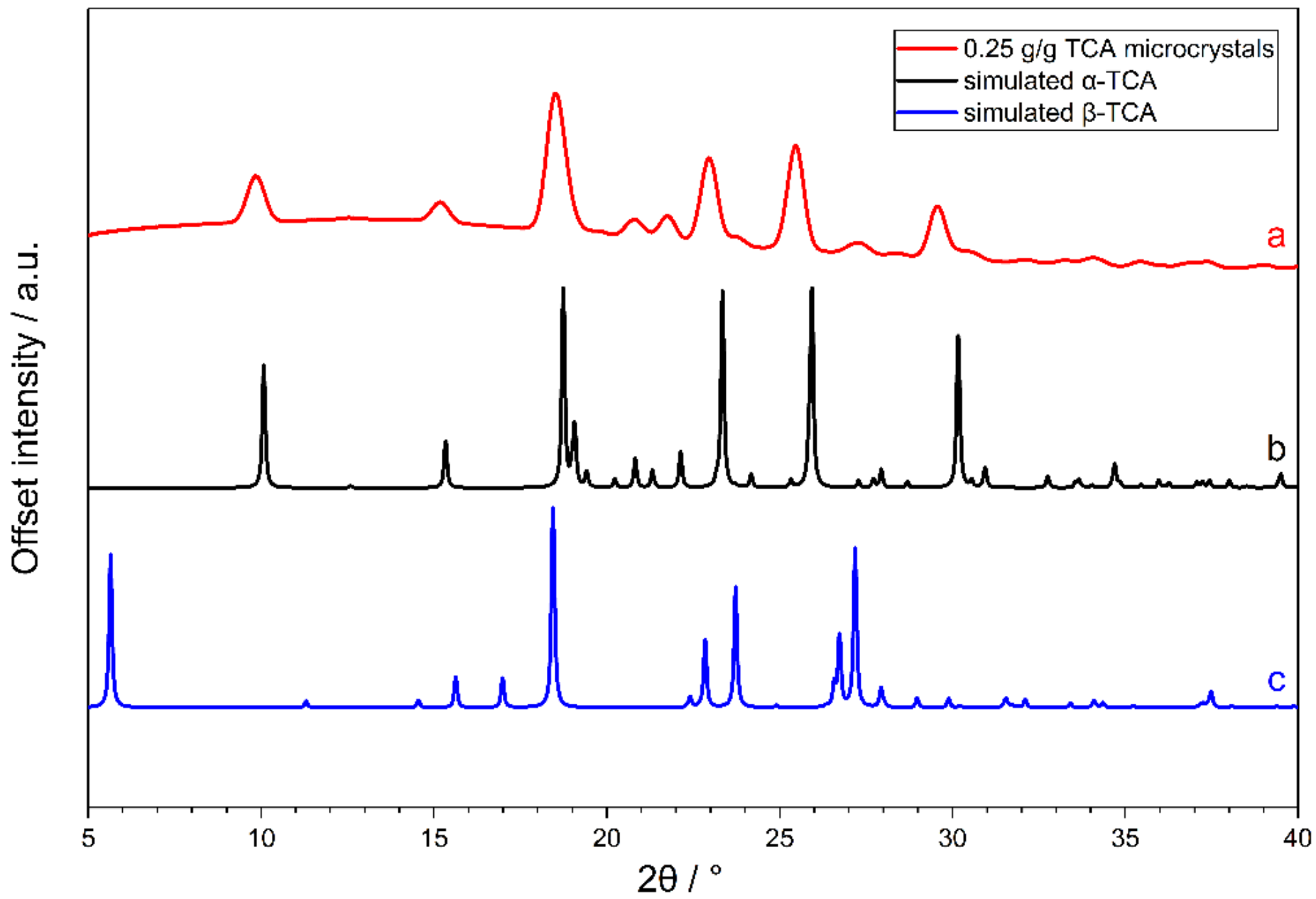

Figure S2 Ex-situ analysis of α-trans-cinnamic acid (TCA) microcrystals obtained by rapid reverse antisolvent crystallisation under continuous sonication using an acetone/light petroleum solvent–antisolvent system (initial TCA concentration: 25 wt% [0.25 g/g]). **(a) FT-IR spectrum of dried crystals** (key vibrations, cm^{-1})**:** 3089, 3067, 3030 [*v*(C-H)], 1668 (s) [*v*(C=O)], 1627 (s) [*v*(C=C) aliphatic], 1575 [*v*(C=C), aromatic], 1494, 1448, 1416, 1311, 1280, 1217, 1177, 1074 [*δ*(C-H) and *v*(C-O)], **(b) PXRD pattern of the dried TCA microcrystalline product (red),** compared with simulated PXRD patterns generated from single-crystal X-ray structures of the known TCA polymorphs: α-TCA (black; CSD refcode: CINMAC07) and β-TCA (blue; CSD refcode: CINMAC06).

Section 2: smSSX photocrystallography data collection

PORTO laser set-up and parameters

Photocrystallographic experiments on α-TCA were performed using the PORTO pulsed femtosecond laser system, described in detail elsewhere.[8, 9] Only parameters relevant to the present study are summarised here. Photoactivation was carried out using the second harmonic of the fundamental laser wavelength, centred at 515 nm. The laser was operated at a repetition rate of 50 kHz and an average power of 3 mW at the sample position and an energy of 0.06 μJ per pulse.

The frequency-doubled beam was directed to the sample position via a series of mirrors and focused using a plano-convex anti-reflection-coated UV-grade fused silica lens (f = 40 cm). At the sample position, the laser beam exhibited full width at half maximum (FWHM) dimensions of 80 μm × 70 μm (horizontal × vertical), as determined by knife-edge measurements using a photodiode. The spatial overlap between the laser and X-ray beams was confirmed using a YAG:Ce screen, with the two beams intersecting at an angle of approximately 10° in the vertical plane. The final steering mirror prior to the fixed-target grid was mounted on a motorised stage equipped with a picomotor, and a pick-off window directed a small fraction of the reflected beam to a camera, enabling monitoring and correction of beam position throughout the experiment.

Laser irradiation of the crystals was controlled using a fast optical shutter (Thorlabs SH1/M) positioned upstream of the sample. Shutter opening periods of 0, 0.5, 2, 7, 10, 20, 100, and 200 s corresponded to delivery of 0, 25000, 100000, 350000, 500000, 1000000, 5000000, and 10000000 laser pulses, respectively. The average laser power at the sample was measured using a calibrated power meter (Thorlabs S401C). The effective irradiation time was estimated as the product of the laser pulse duration (300 fs) and the total number of pulses delivered.

It should be noted that irradiation under these conditions is *not* equivalent to continuous illumination over the same time period: the pulsed nature of the femtosecond PORTO laser beam means that, even though the total irradiation time sums to only 2.25 μs at the longest exposure, each 300 fs pulse delivers its energy in an extremely short interval, producing a peak intensity many orders of magnitude higher than a continuous source with the same average power.

SR-FX-SSX data collection set-up

SSX data were collected in Experimental Hutch 2 of Beamline I19 at Diamond Light Source using a Newport four-circle diffractometer equipped with a Dectris Eiger2 4M CdTe detector positioned at 85 mm. Measurements were performed at the Ag K-edge (λ = 0.4859 Å, 25.5140 keV), enabling atomic resolution data (to < d_{min}= 0.6 Å) to be obtained from a single detector position (single value of 2θ). A SmarAct SLC 2430 XYZ linear piezo stage was mounted on the diffractometer φ axis, allowing fast and reproducible translation between grid wells. Fixed-target grids were prepared as described in Section 1 and mounted on the goniometer using a modified magnetic base with a tweezer-style holder to secure the fixed-target grid. Grids were aligned such that they were approximately normal to both the incident X-ray beam and the on-axis viewing system. Data acquisition was controlled using a custom graphical user interface developed at Beamline I19, as previously described in our work detailing the SR-FT-SSX methodology.[3]

smSSX ground-state data collection protocol

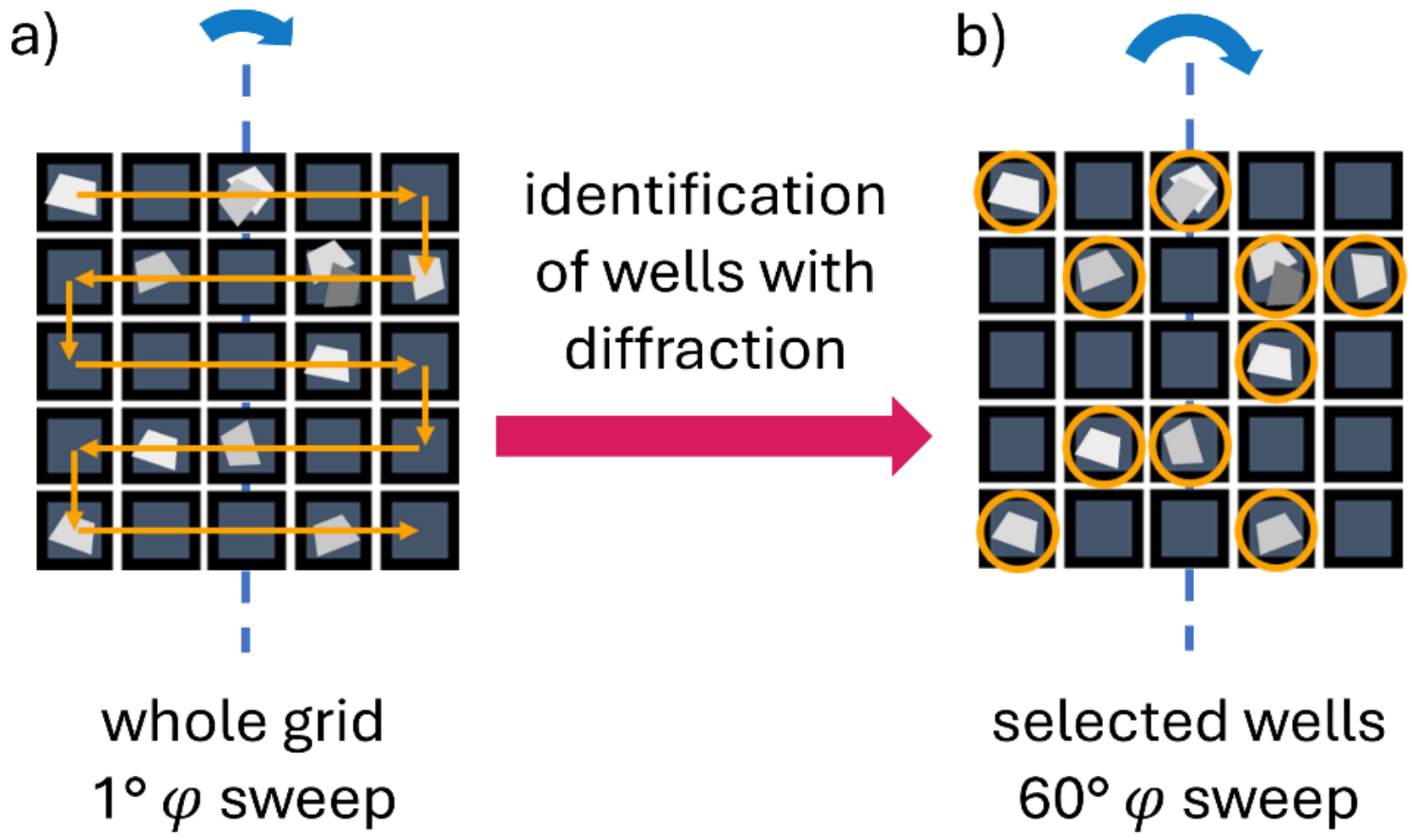

Figure S3 Schematic illustration of the two-part smSSX data-collection protocol. (a) Step one: rapid small-angle (1°) raster scan of the complete fixed-target grid. (b) Step two: wide-angle (60°) data collection from selected well positions.

Photocrystallographic data collections require acquisition of a high-quality ground-state structure to enable reliable comparison with photoexcited datasets and the construction of accurate photodifference maps.[10] In SSX photocrystallography, this necessitates collection of the ground-state data under the same conditions, i.e. ideally using the same fixed-target grid and well positions) as are subsequently used for photoexcited measurements, prior to laser irradiation. To achieve this efficiently, an optimised two-part data-collection strategy was employed, comprising (1) a rapid small-angle whole-grid screening ("spot-finding") scan to identify well positions containing suitable crystals, followed by (2) wide-angle data collection at the selected well positions. This approach minimised dead-time and overheads associated with the unnecessary data collection from empty wells or poorly diffracting crystals, which is an especially important consideration for photocrystallographic experiments in which multiple datasets are collected repeatedly following laser excitation to monitor reaction progression.

For the initial screening step (step one in Figure S3), the fixed-target grid was rastered across all 400 well positions. At each well, diffraction data were collected over a narrow 1° φ-rotation using a 0.2° image width, yielding five diffraction images per position. A low X-ray beam transmission of 5% and an exposure time of 0.1 s per image were used to minimise radiation dose while retaining sufficient signal for crystal identification and preliminary diffraction quality assessment. These step one partial datasets were not subjected to full integration; instead, Bragg peak identification was performed using the DIALS routine *dials.find_spots*,[11] and the number of reflections detected per well was used as the selection criterion. Well positions exhibiting more than 10 Bragg reflections were retained for subsequent wide-angle data collection.

In the second stage (step two in Figure S3), wide-angle datasets were collected from the selected well positions to generate the ground-state X-ray structure (condition 1). At each position, the sample was rotated over a total φ range of 60° with a 0.2° image width, resulting in 300 diffraction images per well. An X-ray beam transmission of 40% and exposure time of 0.2 s per image were identified as optimal during initial testing, yielding high-quality diffraction to a resolution of 0.8 Å. Although this wide-angle approach involves substantially larger rotation ranges than those used in our earlier implementations of the SR-FT-SSX method,[3] it reduced the number of fixed-target grids required, thereby decreasing overall experimental overhead. This strategy was adopted to accommodate beamtime constraints and demonstrates the flexibility of the technique for tailoring data-collection protocols to specific experimental and sample requirements.

Photo-SSX steady-state data collection protocol

Photocrystallographic SR-FT-SSX experiments were conducted by collecting repeated SR-FT-SSX datasets from selected fixed-target well positions following *in situ* laser irradiation immediately prior to X-ray exposure. Targeted irradiation of individual wells was achieved using the PORTO laser setup described above. In total, eight SR-FT-SSX photocrystallographic datasets were collected under different irradiation conditions, including a ground state dataset (Condition 1). Details of all irradiation conditions are summarised in Table 1 of the main manuscript. For each grid, a complete two-step ground-state data collection protocol was performed to identify well positions containing high-quality crystals and to provide a directly comparable ground-state reference structure. Excited-state data collection was carried out sequentially across selected well positions. For example, under Condition 2, each well was irradiated immediately prior to X-ray exposure (laser shutter open time 0.5 s, 25 000 pulses, effective irradiation time 7.5 ns), followed by collection of a wide-angle rotational dataset (total rotation range 60°) using the same protocol as step two of the ground-state SR-FT-SSX procedure described above. This *irradiation* → *data-collection* sequence was then repeated for all selected wells on the grid. The grid was then returned to the initial well position before commencing the next measurement cycle, in which irradiation corresponding to the next experimental condition (Condition 3) was delivered. Conditions 1 – 6 were measured across a total of two fixed-target grids (grids 1 and 2), while Conditions 7 and 8 were collected on a third grid (grid 3) due to beamtime constraints.

Section 3: smSSX photocrystallography data processing

Data processing scripts

Data processing was performed using a modified version of the semi-automated data-processing pipeline described in our previous report detailing the SR-FT-SSX approach.[3] The pipeline is implemented as a Python (v3.10) script and executed on the Diamond Light Source computing cluster, where it submits bash scripts that invoke DIALS[11] for on-the-fly data processing. Data processing jobs are executed in parallel such that, as data collection from an individual well position on the FT grid is completed, the corresponding partial dataset is automatically submitted to the cluster queue and processed as soon as computational resources become available. Upon completion of data collection and processing across the grid, a .csv summary file is generated for downstream analysis. Two key modifications were introduced relative to the original pipeline. First, a rapid, standalone peak-finding mode was implemented for processing of the data collected in step one of the data collection protocol, which identifies and reports the number of Bragg reflections without performing full indexing, integration, scaling, and merging. This substantially reduces computation time and avoids failures that can occur during full processing of datasets collected over very small φ-rotation ranges. Second, inspection of the data revealed that many well positions contained multiple crystals, such that conventional single-lattice indexing captured only a subset of the observed Bragg reflections. To address this, a multi-lattice indexing strategy was implemented,[12] enabling the identification and processing of additional lattices present within a single partial dataset. This multi-lattice indexing approach is described in the following sections.

smSSX multi-lattice indexing approach

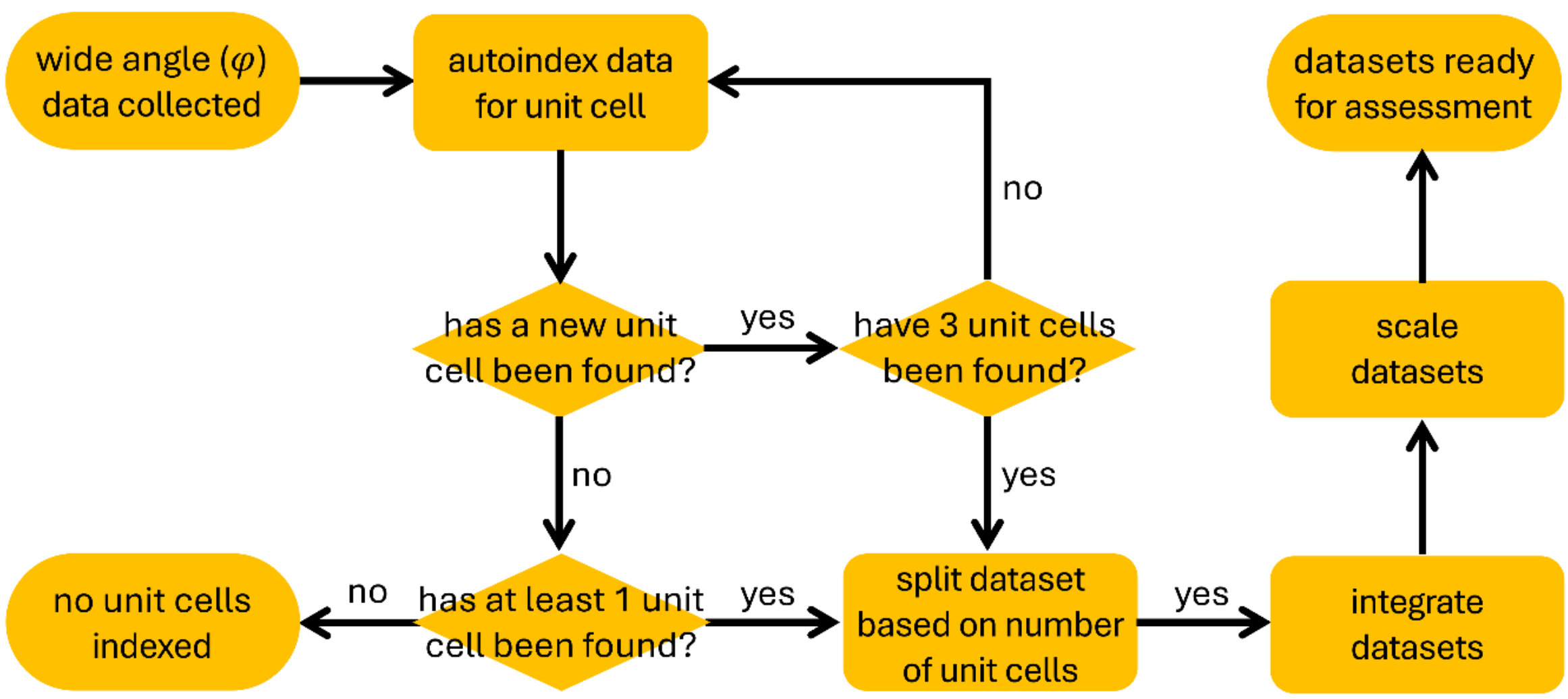

Figure S4 Schematic workflow illustrating the sequence of steps implemented in the multi-lattice auto-indexing strategy.

DIALS commands for multi-lattice autoindexing

```
cd <processing_directory >
module load dials
dials . import < path_to_diffraction_images > \
reference_geometry =< path_to_ref_geom_file > \
mask =< path_to_mask_file >
dials.find_spots imported . expt d_min=0.8
dials.index imported.expt strong.refl \
indexing.method=fft3d \
max_lattices=3 refinement.parameterisation.beam.fix=all \
refinement.parameterisation.detector.fix=all \
refinement_protocol.d_min_final=0.8
dials.split_experiments indexed.expt indexed.refl
if grep -q 'model 1' 'dials.index.log '; then
        mkdir split_0
        cp split_0.{expt,refl} split_0/
        cd split_0/
        dials.refine split_0.expt split_0.refl outlier.algorithm=tukey
        dials.integrate refined.expt refined.refl min_spots.overall=1 \
        min_spots.per_degree=1 prediction.d_min=0.8
        dials.two_theta_refine integrated.expt integrated.refl
        dials.scale refined_cell.expt integrated.refl d_min=0.8
        cd ..
fi
if grep -q 'model 2' 'dials.index .log '; then
        mkdir split_1
        cp split_1.{expt,refl} split_1 /
        cd split_1 /
        dials.refine split_1.expt split_1.refl outlier.algorithm=tukey
        dials.integrate refined.expt refined.refl min_spots.overall =1 \
        min_spots.per_degree=1 prediction.d_min=0.8
        dials.two_theta_refine integrated.expt integrated.refl
        dials.scale refined_cell.expt integrated.refl d_min=0.8
        cd ..
fi
        if grep -q 'model 3' 'dials.index .log '; then
        mkdir split_2
        cp split_2.{expt,refl} split_2 /
        cd split_2 /
        dials.refine split_2.expt split_2.refl outlier.algorithm=tukey
        dials.integrate refined.expt refined.refl min_spots.overall=1 \
        min_spots.per_degree=1 prediction.d_min=0.8
        dials.two_theta_refine integrated.expt integrated.refl
        dials.scale refined_cell.expt integrated.refl d_min=0.8
fi
```

Indexing of unit cell parameters from photo- SSX datasets via multi-lattice autoindexing

A key feature of the multi-lattice auto-indexing mode is the introduction of an iterative indexing procedure (as described in Figure S4),[12] which is designed to account for cases in which multiple crystals occupy a single well of the fixed-target grid. In contrast to the single-lattice auto-indexing approach used previously,[3] each partial dataset is indexed multiple times in an effort to identify additional unit cells arising from the presence of multiple crystals. In this study, up to three distinct unit cells were permitted to be indexed from a single partial dataset, thereby increasing the amount of usable data obtainable from the wide-angle (step two) measurements by up to a factor of three. This upper limit was selected to minimise the identification of unit cells derived from an insufficient number of Bragg reflections and therefore considered unreliable. Auto-indexing was performed using the FFT3D algorithm; this approach was chosen in preference to the RSGS routine used in our earlier study of sodium nitroprusside dihydrate[3] because the unit-cell parameters of α-TCA are known to vary significantly during the photo-induced reaction.[13] Accordingly, no reference unit cell was supplied in order to avoid bias in the determined cell parameters. During partial dataset processing, a decrease in diffraction strength, as assessed by $\langle I/\sigma(I)\rangle$ and the maximum achievable resolution, was initially observed with continued laser irradiation for datasets collected under Conditions 2 – 6, followed by partial recovery for Conditions 7 and 8 at longer irradiation times. On this basis, condition-specific resolution cut-offs were selected and applied uniformly to all partial datasets collected under each irradiation condition.

Statistical analysis of unit cell parameter spread as a function of laser exposure

Following completion of the multi-lattice indexing routines, partial datasets were filtered based on structure-quality indicators to exclude weak or unreliable solutions. Specifically, only partial datasets satisfying $\langle I/\sigma(I)\rangle \geq 1.5$ and $0 < R_{pim} \leq 0.2$ were retained, thereby removing partial datasets that were likely to have been incorrectly indexed. The filtered partial datasets were then analysed by plotting the β angle as a function of the logarithm of the number of laser pulses delivered to the corresponding well positions by the PORTO laser. The data are presented as box-and-whisker plots in Figure 3a of the main manuscript, from which significant outliers have been removed for clarity. The unfiltered box-and-whisker plot is shown in Figure S5, together with the corresponding summary statistics listed in Table S2.

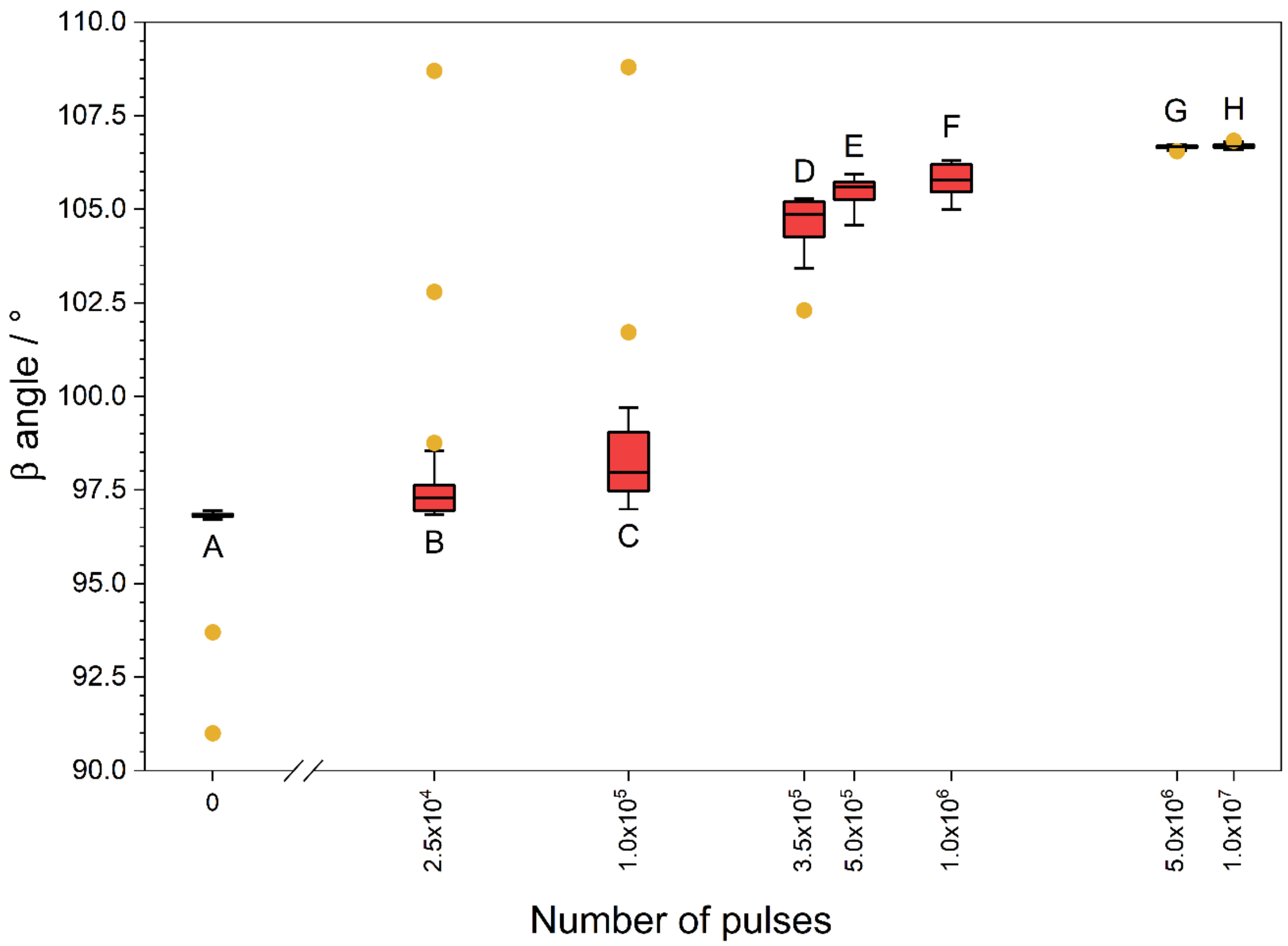

Figure S5 Box and whisker plot showing the spread of *β* angles across the microcrystal batch with all data points included, including outlier datasets omitted from Figure 3a in the main manuscript (yellow = outlier datasets).

Table S2 Statistical summary of *β* angles obtained by FFT3D auto-indexing from all selected partial datasets collected under each photo-SSX data-collection condition.

Condition #	Number of datasets	Median	First quartile	Third quartile	Upper whisker	Lower whisker	Interquartile range
A	88	96.817	96.795	96.858	96.947	96.72	0.063
B	33	97.299	96.95	97.63	98.55	96.839	0.68
C	18	97.98	97.48	99.04	99.7	96.99	1.56
D	9	104.86	104.27	105.212	105.28	103.43	0.942
E	16	105.595	105.265	105.725	105.94	104.58	0.46
F	6	105.79	105.46	106.198	106.3	105	0.738
G	48	106.673	106.644	106.696	106.74	106.575	0.052
H	45	106.69	106.657	106.723	106.8	106.6	0.066

smSSX partial dataset filtering and selection using dials.cosym

The substantial spread observed in the indexed unit cell parameters indicates that the population of the photo-induced excited state varies significantly across the microcrystal batch, even under identical irradiation conditions (Figure S5). The underlying reasons for this batch-scale behaviour are discussed in detail in the main manuscript. Consequently, a diffraction data-based strategy was adopted for the selection of datasets suitable for scaling and merging into a final combined serial crystal structure for each irradiation condition, using the *cosym* program within the DIALS processing suite.[14] Although the β unit cell angle is most often used as the primary metric to monitor the progression of the [2+2] photodimerization reaction, the remaining unit cell parameters were also found to vary. Dataset selection therefore could not be based solely on the β angle and instead required consideration of all unit cell parameters to ensure structural comparability. To this end, all datasets successfully processed by the multi-lattice auto-indexing procedure, as described above, were analysed using *cosym* to determine median unit cell parameters for each irradiation condition, which are reported in Table S3. The corresponding raw dials.cosym outputs are provided in Figures S6 – S13.

Following determination of the median unit cell parameters for each irradiation condition, dials.cosym was used to perform hierarchical clustering of the unit cell parameters within each condition to identify partial datasets most suitable for scaling and merging. Partial datasets suitable for inclusion in the final combined serial structures were selected based on their consistency with the median lattice determined for the corresponding irradiation condition, thereby improving the robustness of downstream data processing. Final dataset selection was based on satisfying the criteria $\langle I/\sigma(I)\rangle \geq 2$ and unit cell parameters within ±0.5 Å for cell lengths and ±0.5° for cell angles relative to the median values determined by dials.cosym. The resulting filtered datasets for each irradiation condition were then scaled and merged using dials.scale, employing parameters appropriate for small-rotation multi-crystal datasets, as described in our original SR-FT-SSX methodology report.[3] The final output comprised eight combined serial datasets, one corresponding to each irradiation condition, which were used for subsequent structure solution and refinement as described in ESI Section 4.

Table S3 Median unit cell parameters output by dials.cosym for conditions 1 – 8. Values of α and γ were constrained to 90° by the *cosym* program following correct identification of the *P*12/*m*1 Patterson symmetry.

Condition	*a* / Å	*b* / Å	*c* / Å	*β* / °
1	5.637	17.968	7.744	96.823
2	5.635	18.051	7.737	97.279
3	5.638	18.289	7.692	99.714
4	5.694	18.924	7.584	105.282
5	5.695	18.879	7.602	105.536
6	5.700	18.710	7.640	106.310
7	5.699	18.647	7.665	106.683
8	5.699	18.640	7.661	106.694

```
+-------------------+-----+--------------+----------+--------+--------+---------+--------------------+
| Patterson group   |     |   Likelihood |   NetZcc |   Zcc+ |   Zcc- |   delta | Reindex operator   |
|-------------------+-----+--------------+----------+--------+--------+---------+--------------------|
| P 1 2/m 1         | *** |        0.949 |       10 |     10 |      0 |       0 | -a,-c,-b           |
| P -1              |     |        0.051 |      -10 |      0 |     10 |       0 | -a,-b,c            |
+-------------------+-----+--------------+----------+--------+--------+---------+--------------------+
Best solution: P 1 2/m 1
Unit cell: 5.637, 17.968, 7.744, 90.000, 96.823, 90.000
Reindex operator: -a,-c,-b
Laue group probability: 0.949
Laue group confidence: 0.924
```

Figure S6 dials.cosym output for irradiation condition 1

```
Scoring all possible sub-groups
+-------------------+----+--------------+----------+--------+--------+---------+--------------------+
| Patterson group   |    |   Likelihood |   NetZcc | Zcc+   | Zcc-   |   delta | Reindex operator   |
|-------------------+----+--------------+----------+--------+--------+---------+--------------------|
| P 1 2/m 1         | *  |        0.578 |     7.75 | 7.75   | 0      |       0 | -a,-c,-b           |
| P -1              | *  |        0.422 |    -7.75 | 0      | 7.75   |       0 | -a,-b,c            |
+-------------------+----+--------------+----------+--------+--------+---------+--------------------+
Best solution: P 1 2/m 1
Unit cell: 5.635, 18.051, 7.737, 90.000, 97.279, 90.000
Reindex operator: -a,-c,-b
Laue group probability: 0.578
Laue group confidence: 0.300
```

Figure S7 dials.cosym output for irradiation condition 2

```
Scoring all possible sub-groups
+-------------------+----+--------------+----------+--------+--------+---------+--------------------+
| Patterson group   |    |   Likelihood |   NetZcc | Zcc+   | Zcc-   |   delta | Reindex operator   |
|-------------------+----+--------------+----------+--------+--------+---------+--------------------|
| P -1              | ** |        0.601 |    -6.83 | 0      | 6.83   |       0 | -a,-b,c            |
| P 1 2/m 1         |    |        0.399 |     6.83 | 6.83   | 0      |       0 | -a,-c,-b           |
+-------------------+----+--------------+----------+--------+--------+---------+--------------------+
Best solution: P -1
Unit cell: 5.638, 7.692, 18.289, 90.000, 90.000, 99.714
Reindex operator: -a,-b,c
Laue group probability: 0.601
Laue group confidence: 0.348
```

Figure S8 dials.cosym output for irradiation condition 3

```
Scoring all possible sub-groups
+-------------------+----+--------------+----------+--------+--------+---------+--------------------+
| Patterson group   |    |   Likelihood |   NetZcc | Zcc+   | Zcc-   |   delta | Reindex operator   |
|-------------------+----+--------------+----------+--------+--------+---------+--------------------|
| P -1              | *  |        0.522 |    -7.27 | 0      | 7.27   |       0 | -a,-b,c            |
| P 1 2/m 1         | *  |        0.478 |     7.27 | 7.27   | 0      |       0 | -a,-c,-b           |
+-------------------+----+--------------+----------+--------+--------+---------+--------------------+
Best solution: P -1
Unit cell: 5.694, 7.584, 18.924, 90.000, 90.000, 105.282
Reindex operator: -a,-b,c
Laue group probability: 0.522
Laue group confidence: 0.153
```

Figure S9 dials.cosym output for irradiation condition 4

```
Scoring all possible sub-groups
+-------------------+----+--------------+----------+--------+--------+---------+--------------------+
| Patterson group   |    |   Likelihood |   NetZcc |   Zcc+ |   Zcc- |   delta | Reindex operator   |
|-------------------+----+--------------+----------+--------+--------+---------+--------------------|
| P 1 2/m 1         | ** |         0.61 |      7.9 |    7.9 |    0   |       0 | -a,c,b             |
| P -1              |    |         0.39 |     -7.9 |    0   |    7.9 |       0 | a,b,c              |
+-------------------+----+--------------+----------+--------+--------+---------+--------------------+
Best solution: P 1 2/m 1
Unit cell: 5.695, 18.879, 7.602, 90.000, 105.536, 90.000
Reindex operator: -a,c,b
Laue group probability: 0.610
Laue group confidence: 0.367
```

Figure S10 dials.cosym output for irradiation condition 5

```
Point group    a           b           c          alpha       beta        gamma


1 cluster

Cluster_id       N_xtals  Med_a         Med_b         Med_c         Med_alpha    Med_beta     Med_gamma   Delta(deg)
64 in P 1 21/n 1.
cluster_1        64       5.70  (0.14 ) 18.71 (0.76 ) 7.64  (0.31 ) 90.00 (0.00) 106.31(3.46) 90.00 (0.00)
      P 1 2/m 1 (No. 10)  5.70          18.71         7.64          90.00        106.31       90.00         0.0
Standard deviations are in brackets.
Each cluster:
        Input lattice count, with integration Bravais setting space group.
        Cluster median with Niggli cell parameters (std dev in brackets).
        Highest possible metric symmetry and unit cell using LePage (J Appl Cryst 1982, 15:255) method, maximum delta 3deg.
```

Figure S11 dials.cosym output for irradiation condition 6

```
Scoring all possible sub-groups
+-------------------+-----+--------------+----------+--------+--------+---------+--------------------+
| Patterson group   |     |   Likelihood |   NetZcc |   Zcc+ |   Zcc- |   delta | Reindex operator   |
|-------------------+-----+--------------+----------+--------+--------+---------+--------------------|
| P 1 2/m 1         | *** |        0.943 |     9.85 |   9.85 |   0    |       0 | -a,c,b             |
| P -1              |     |        0.057 |    -9.85 |   0    |   9.85 |       0 | a,b,c              |
+-------------------+-----+--------------+----------+--------+--------+---------+--------------------+
Best solution: P 1 2/m 1
Unit cell: 5.699, 18.647, 7.665, 90.000, 106.683, 90.000
Reindex operator: -a,c,b
Laue group probability: 0.943
Laue group confidence: 0.914
```

Figure S12 dials.cosym output for irradiation condition 7

```
Scoring all possible sub-groups
+-------------------+----+--------------+----------+--------+--------+---------+--------------------+
| Patterson group   |    |   Likelihood |   NetZcc |   Zcc+ |   Zcc- |   delta | Reindex operator   |
|-------------------+----+--------------+----------+--------+--------+---------+--------------------|
| P 1 2/m 1         | ** |        0.727 |     8.41 |   8.41 |   0    |       0 | -a,c,b             |
| P -1              |    |        0.273 |    -8.41 |   0    |   8.41 |       0 | a,b,c              |
+-------------------+----+--------------+----------+--------+--------+---------+--------------------+
Best solution: P 1 2/m 1
Unit cell: 5.699, 18.640, 7.661, 90.000, 106.694, 90.000
Reindex operator: -a,c,b
Laue group probability: 0.727
Laue group confidence: 0.574
```

Figure S13 dials.cosym output for irradiation condition 8

Section 4: Merged serial X-ray structure solution and refinement

smSSX structure solution and refinement details

Final serial crystallography datasets for each irradiation condition, obtained by merging and scaling the selected partial datasets via dials.scale, were solved using dual-space methods in SHELXT[15] and refined by full-matrix least-squares methods on F^2 using SHELXL.[16] For all irradiation conditions, the merged datasets were refined using a single-component structural model, and no explicit disorder modelling of intermediate reaction states was applied.

This approach was adopted due to the progressive [2+2] cycloaddition reaction, which gives rise to mixed or intermediate average crystal structures and is associated with a systematic reduction in the maximum achievable diffraction resolution. Specifically, the maximum resolution decreases from 0.80 Å for condition 1 to approximately 1.20 Å for conditions 2–6. At higher irradiation doses corresponding to conditions 7 and 8, where photoconversion approaches 100%, the maximum resolution recovers to 0.80 Å as crystallographic disorder is reduced.

The reduction in resolution for the intermediate irradiation conditions resulted in fewer partial datasets meeting the quality criteria required for merging (as detailed in Section 3), thereby lowering the effective data-to-parameter ratio for these structures. Under such conditions, modelling the reaction intermediates using a two-component disorder model would have necessitated an excessive number of parameters relative to the available observational data, leading to over-parameterised and physically non-meaningful refinements. Consequently, disorder modelling was deemed inappropriate for these datasets.

Although disorder models of intermediate photochemical states have been reported previously, such models have typically been limited to partitioning the alkene carbon atoms between the monomeric double bond and the cyclobutane ring of the photodimer.[17] Importantly, these approaches are an approximation as they neglect concomitant structural rearrangements elsewhere in the molecule, particularly the displacement of the phenyl ring and carboxylic acid moieties required to accommodate cyclobutane formation during the reaction. Similarly, although the acidic hydrogen atom was observed to be distributed between the two oxygen atoms of the carboxylic acid group, consistent with static, statistical disorder of the COOH moiety throughout the crystal, the limited data quality of the intermediate irradiation datasets precluded the consistent modelling of this feature for all structures. To ensure uniform refinement protocols and enable direct comparison across all irradiation conditions, this disorder was therefore not modelled for any dataset.

Given that all structures across the full range of irradiation conditions refined successfully using stable weighting schemes within a single-component model, this strategy was considered the most appropriate. It ensures internally consistent refinements and enables direct structural comparison between datasets obtained at different stages of the photoreaction.

Table S4 Crystal data tables summarising the full crystal structure data for the final merged serial datasets for each irradiation condition **1 – 8**.

Condition #	1	2	3	4	5	6	7	8
CCDC deposition #	2554301	2554298	2554300	2554297	2554296	2554295	2554302	2554299
# of merged datasets	63	26	6	23	15	8	48	47
Empirical formula	$C_9H_8O_2$	$C_9H_8O_2$	$C_9H_8O_2$	$C_9H_8O_2$	$C_9H_8O_2$	$C_9H_8O_2$	$C_9H_8O_2$	$C_9H_8O_2$
Formula weight	148.15	148.15	148.15	148.15	148.15	148.15	148.15	148.15
Wavelength / Å	0.4859	0.4859	0.4859	0.4859	0.4859	0.4859	0.4859	0.4859
Temperature / K	293	293	293	293	293	293	293	293
Crystal system	Monoclinic	Monoclinic	Monoclinic	Monoclinic	Monoclinic	Monoclinic	Monoclinic	Monoclinic
Space group	$P2_1/n$	$P2_1/n$	$P2_1/n$	$P2_1/n$	$P2_1/n$	$P2_1/n$	$P2_1/n$	$P2_1/n$
a / Å	5.63589(7)	5.6344(4)	5.605(5)	5.678(2)	5.683(2)	5.714(2)	5.6942(2)	5.6936(2)
b / Å	17.9691(3)	18.009(2)	18.56(4)	18.918(7)	18.820(7)	18.690(7)	18.649(1)	18.643(1)
c / Å	7.74230(9)	7.7343(5)	7.625(9)	7.562(3)	7.600(3)	7.630(3)	7.6595(3)	7.6570(3)
β / °	96.827(1)	97.022(6)	100.0(1)	105.27(3)	105.72(3)	106.31(3)	106.675(3)	106.695(3)
Volume	778.52(2)	778.9(1)	781(2)	783.5(5)	782.5(5)	782.1(5)	779.14(6)	778.47(6)
Z	4	4	4	4	4	4	4	4
ρ_{calc}	1.264	1.263	1.259	1.256	1.258	1.258	1.263	1.264
μ / mm^{-1}	0.044	0.044	0.044	0.044	0.044	0.044	0.044	0.044
F(000)	312	312	312	312	312	312	312	312
Total reflections	88838	36085	2254	7730	9679	1777	68012	63627
Unique reflections	1594	1604	445	473	821	457	1595	1594
Mean $I/\sigma(I)$	64.0	24.5	7.2	10.4	12.8	2.6	43.4	43.9
R_{int}	0.104	0.155	0.126	0.384	0.226	0.848	0.122	0.122
R_{merge}	0.105	0.157	0.136	0.386	0.337	0.749	0.125	0.083
R_{meas}	0.106	0.161	0.157	0.4	0.351	0.861	0.126	0.085
R_{pim}	0.014	0.035	0.074	0.1	0.099	0.416	0.02	0.013
$CC_{1/2}$	1.000	0.995	0.989	0.998	0.999	0.712	1.000	1.000
Completeness θ_{max} (θ_{full}) / %	99.9 (99.9)	99.8 (99.9)	93.5 (93.5)	99.8 (99.8)	99.6(99.6)	96.0 (96.0)	99.9 (99.9)	99.9 (99.9)
Multiplicity	55.73	22.50	5.07	16.34	11.79	3.89	42.64	39.92
d_{max} / Å	0.8	0.8	1.2	1.2	1	1.2	0.8	0.8
R_1 [I>=2$\sigma(I)$]	0.0483	0.0576	0.0728	0.0711	0.0788	0.0491	0.0496	0.0495
wR_2 (all data)	0.1565	0.1770	0.2106	0.2196	0.2352	0.1126	0.1527	0.1536
GooF on F^2	1.086	0.930	0.825	1.052	0.981	1.015	1.118	1.082
Largest peak/hole eÅ^{-3}	0.23/-0.24	0.20/-0.13	0.21/-0.15	0.27/-0.13	0.41/-0.24	0.12/-0.12	0.17/-0.15	0.15/-0.17

Final combined serial crystal structures

Figures S14 – S21 show the atomic connectivity in the final merged serial crystal structures generated for each irradiation condition. The figures show a representative pair of nearest neighbour TCA molecules, arranged head-to-tail enabling [2+2] photodimerization to occur. The labelled non-hydrogen atoms show the asymmetric unit of the crystal structure in each case.

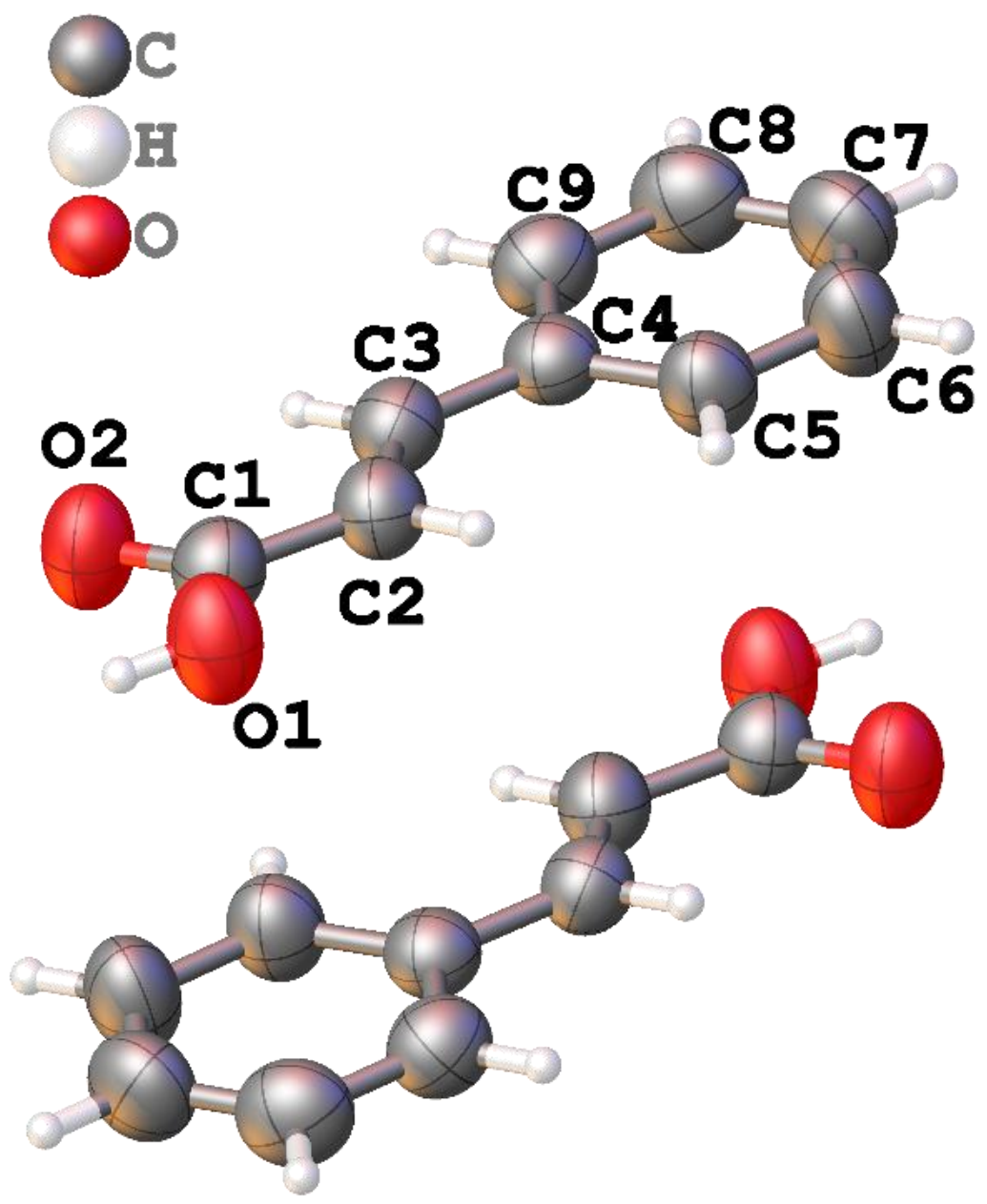

Figure S14 Serial crystal X-ray structure under condition 1 (# laser pulses, *n* = 0) showing the ground state (dark) structure of α-TCA prior to irradiation, viewed along [100]. The intermolecular distance between head-to-tail arranged TCA molecules is 3.660(2) Å, measured as the interplanar distance between aligned C(2)=C(3) moieties in the central and symmetry-related [1-X, 1-Y, 1-Z] neighbouring molecule shown.

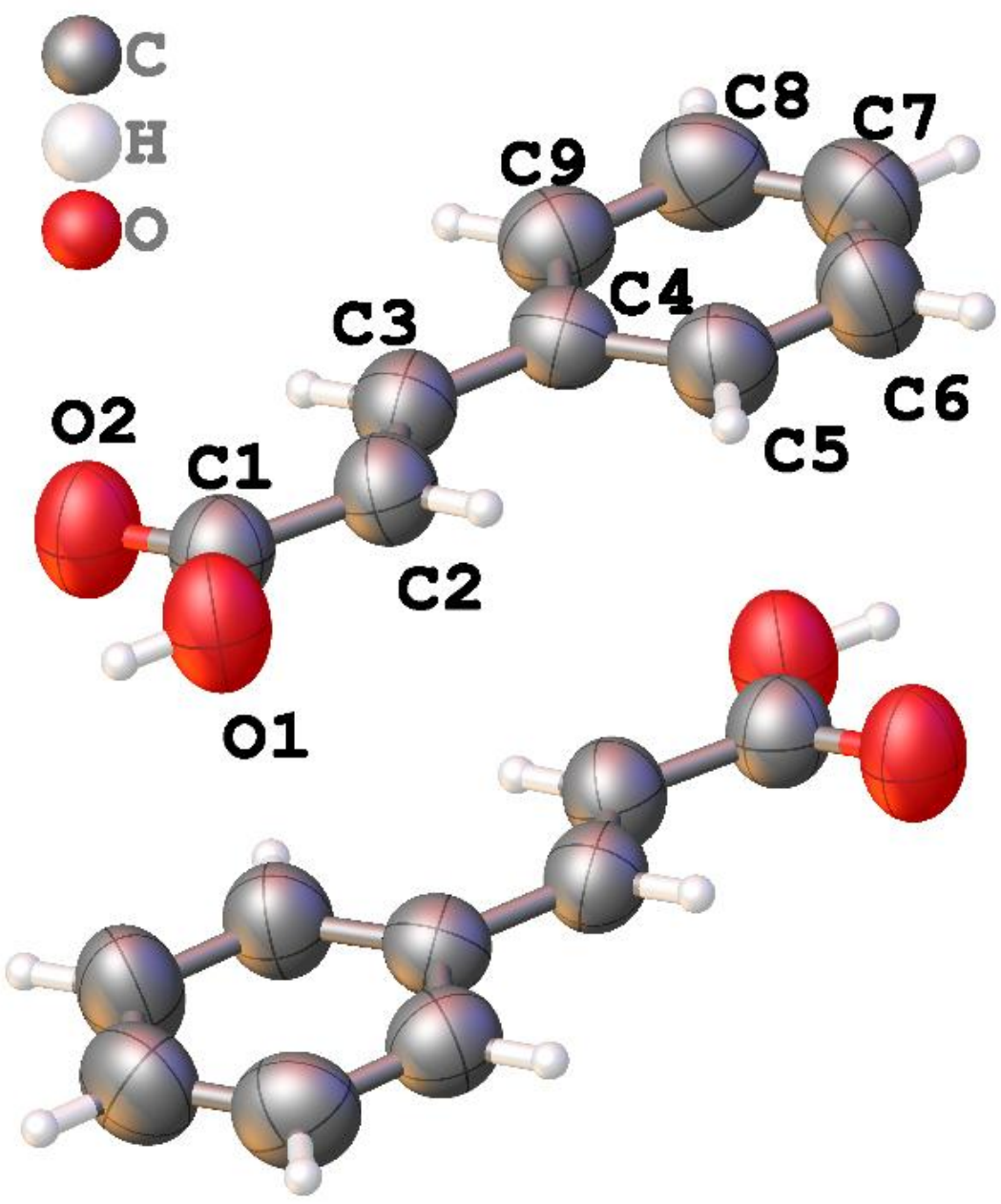

Figure S15 Serial crystal X-ray structure under condition 2 (# laser pulses, *n* = 2.5 x10^{4}) showing the structure of α-TCA prior after 7.5 ns of total laser exposure, viewed along [100].

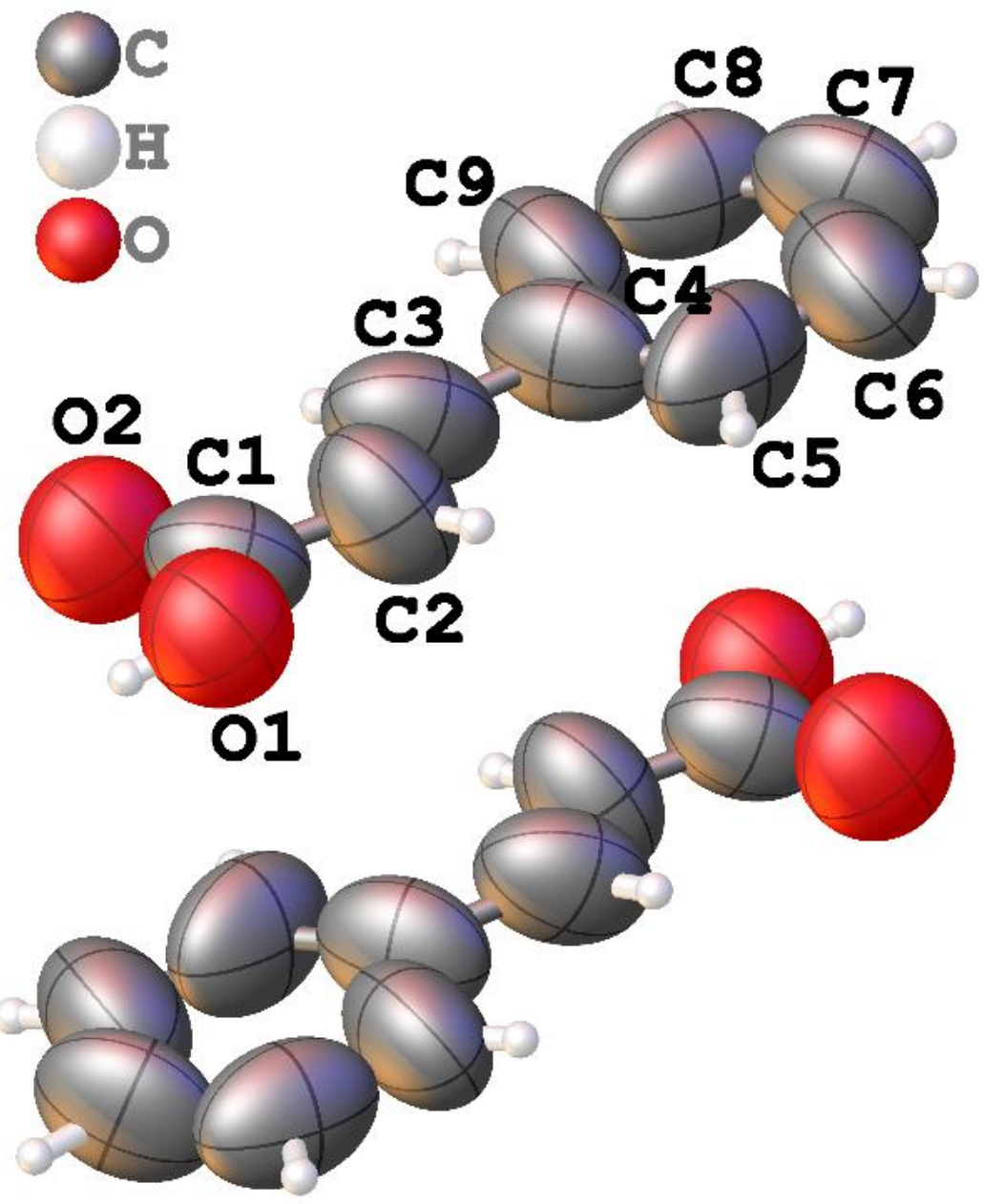

Figure S16 Serial crystal X-ray structure under condition 3 (# laser pulses, *n* = 1.0 x10^5) showing the structure of α-TCA prior after 30 ns of total laser exposure, viewed along [100].

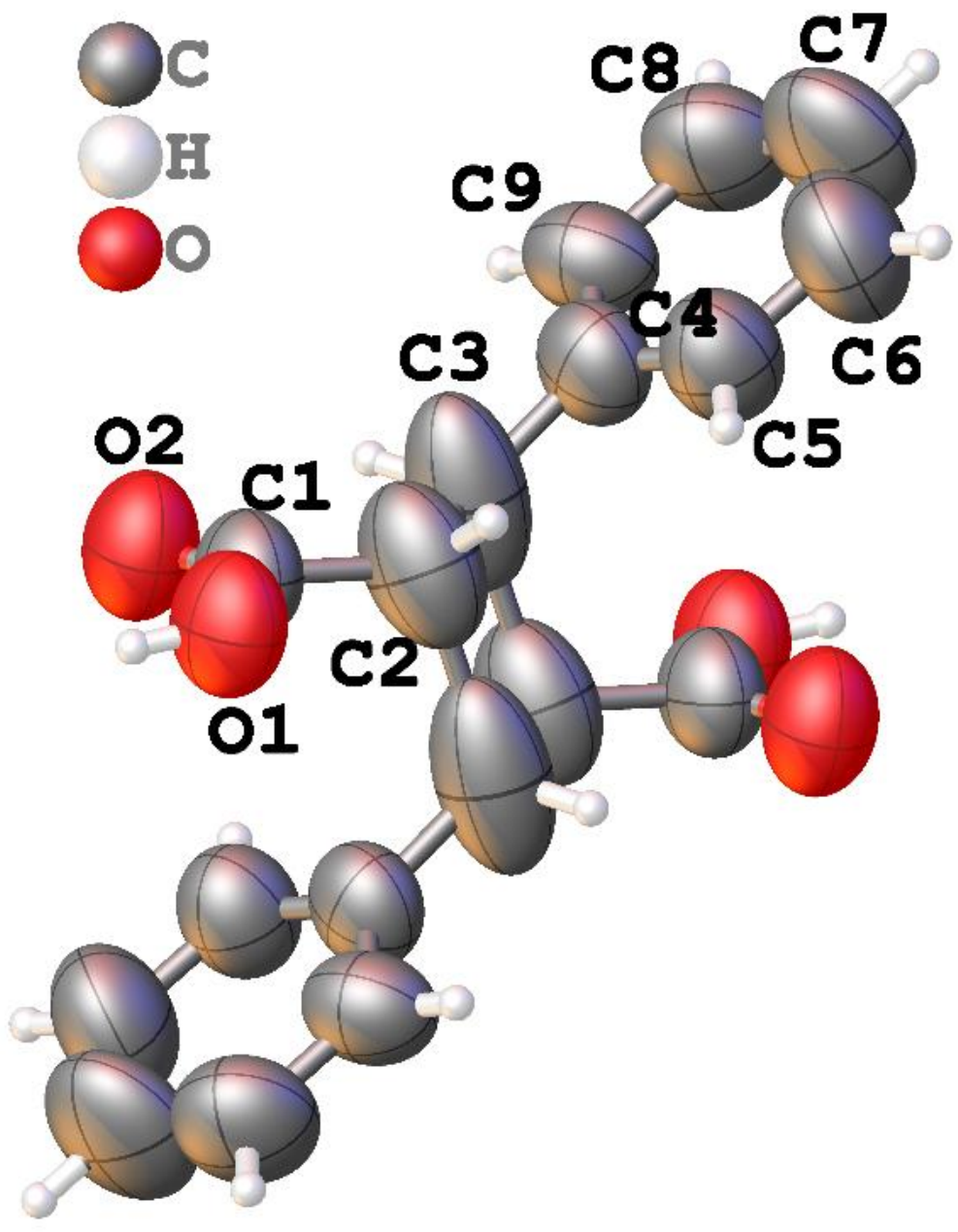

Figure S17 Serial crystal X-ray structure under condition 4 (# laser pulses, *n* = 3.5 x10^5) showing the structure of α-TCA prior after 105 ns of total laser exposure, viewed along [100].

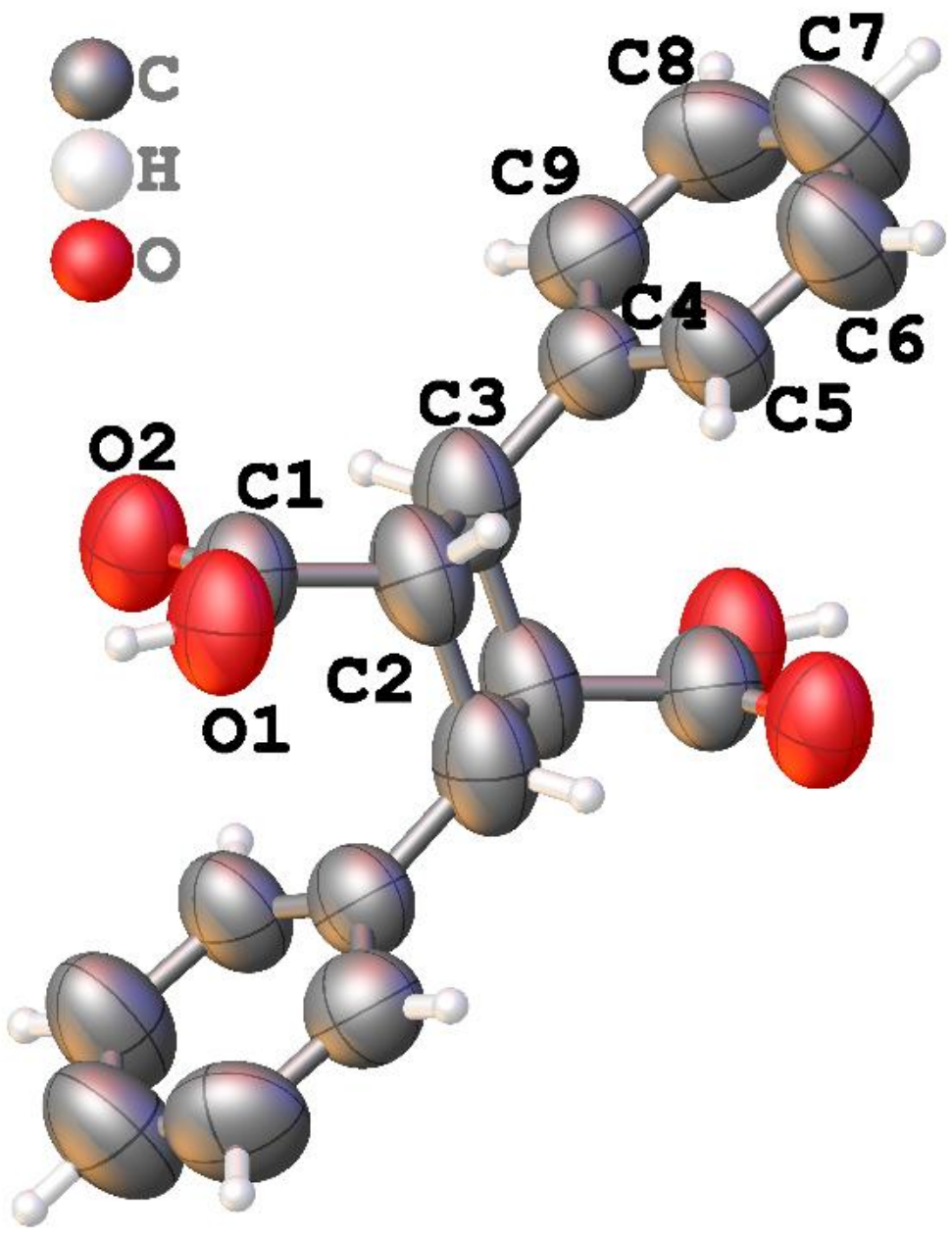

Figure S18 Serial crystal X-ray structure under condition 5 (# laser pulses, *n* = 5.0 x10^{5}) showing the structure of α-TCA prior after 150 ns of total laser exposure, viewed along [100].

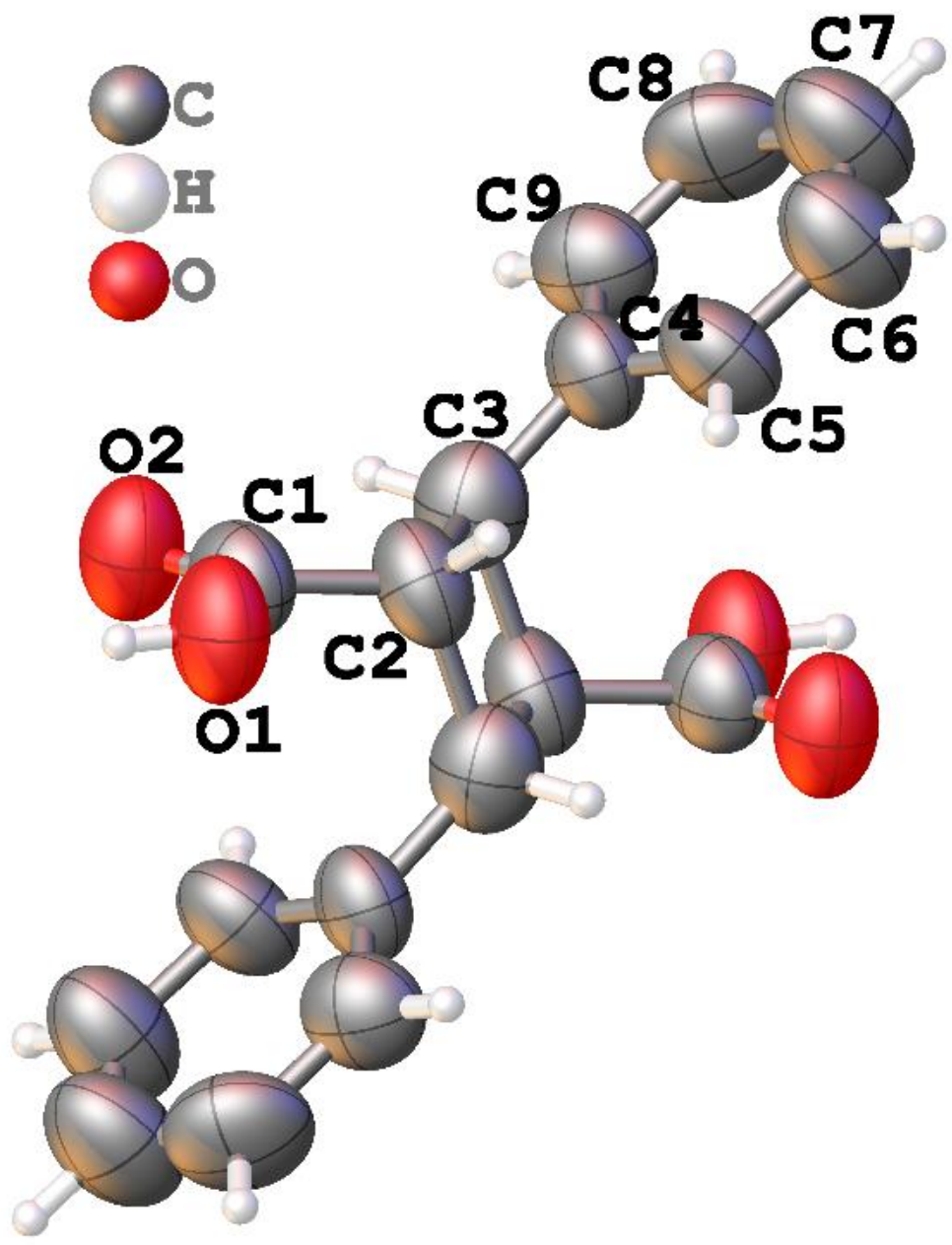

Figure S19 Serial crystal X-ray structure under condition 6 (# laser pulses, *n* = 1.0 x10^{6}) showing the structure of α-TCA prior after 300 ns of total laser exposure, viewed along [100].

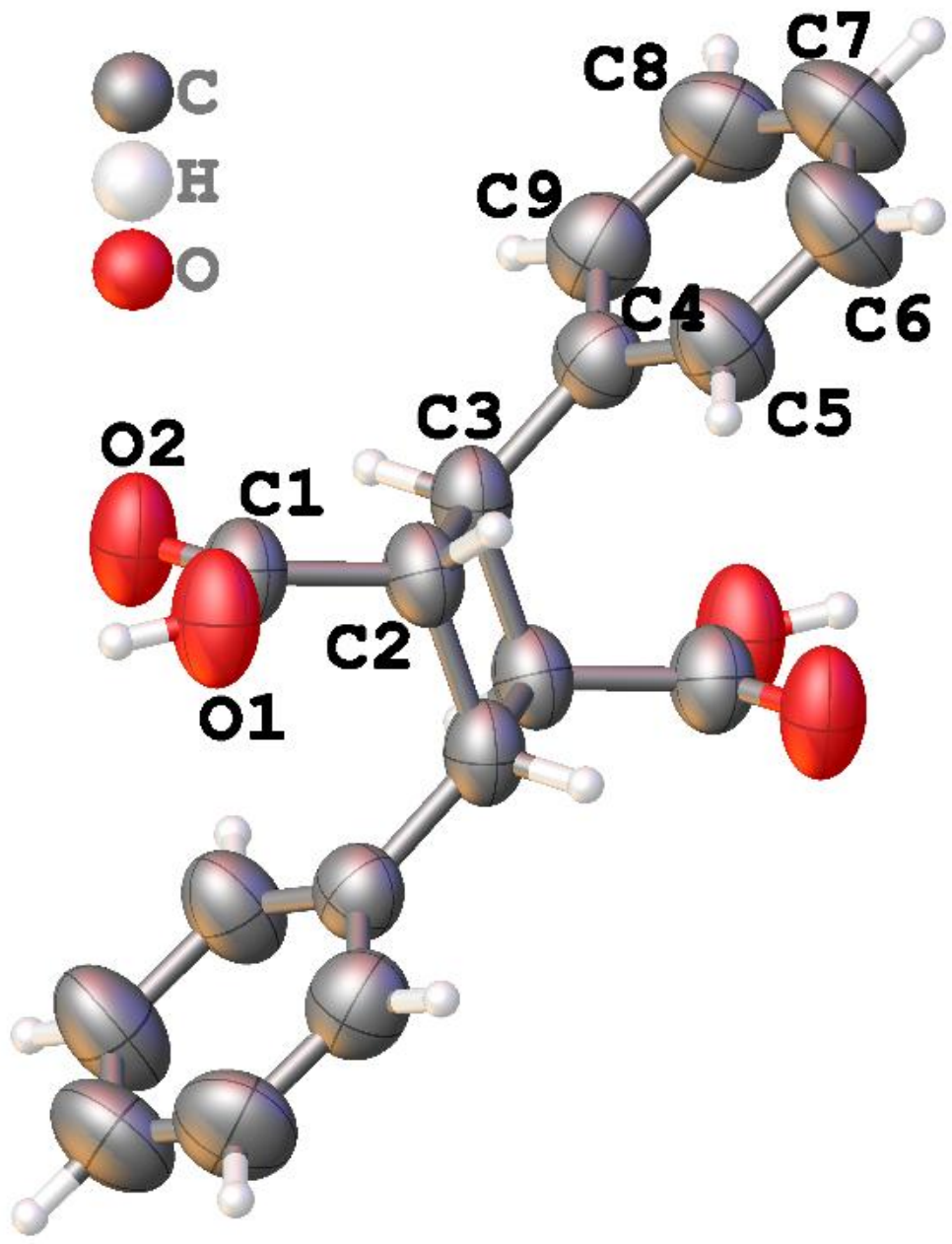

Figure S20 Serial crystal X-ray structure under condition 7 (# laser pulses, *n* = 5.0 x10[6]) showing the structure of α-TCA prior after 1500 ns of total laser exposure, viewed along [100].

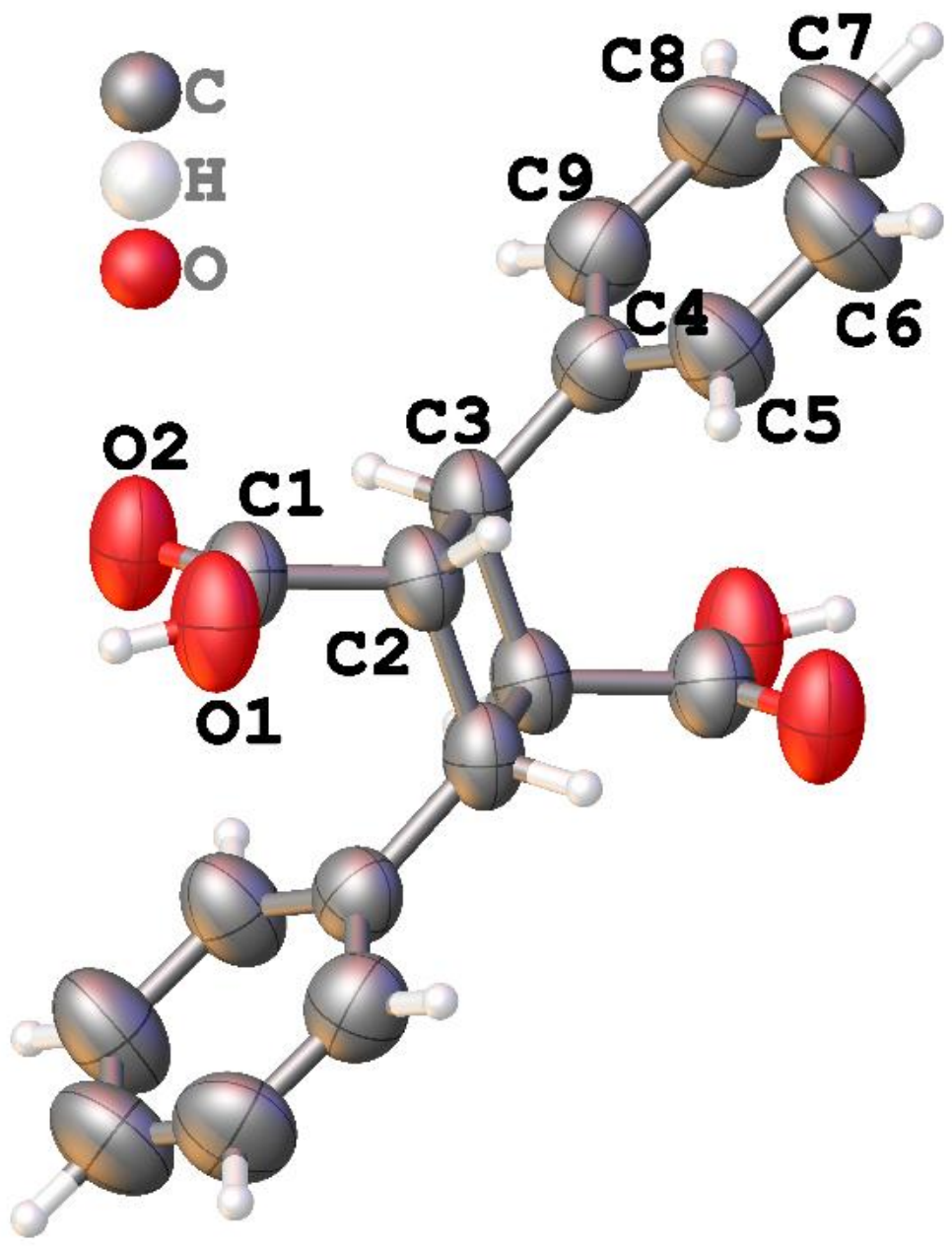

Figure S21 Serial crystal X-ray structure under condition 8 (# laser pulses, *n* = 1.0 x10[7]) showing the structure of α-TCA prior after 3000 ns of total laser exposure, viewed along [100].

Section 5: Validation Analyses

5.1 Sensitivity analysis for partial dataset filtering thresholds

A sensitivity analysis was performed to assess whether the empirically selected thresholds for partial dataset filtering (see **smSSX partial dataset filtering and selection using dials.cosym**, Section 3) influenced the structural parameters obtained. The partial datasets from Condition 3 were reprocessed using three filtering thresholds, retaining datasets with unit cell parameters within (i) ±0.3 Å/°, (ii) ±0.5 Å/° (default value), or (iii) ±0.7 Å/° of the median values. The resulting dataset subsets were independently scaled, merged, solved and refined. As shown in Table S5, progressively relaxing the filtering criteria increased the number of partial datasets/observations retained, resulting in higher completeness, multiplicity and total reflection counts. However, this did not result in systematically improved residual factors. Importantly, variation of the filtering threshold produced only negligible changes in the unit cell parameters, particularly β, and does not affect the structural interpretation. These results demonstrate that the reported conclusions are robust to moderate variation in the partial dataset filtering criteria.

Table S5: Sensitivity analysis for partial dataset filtering of Condition 3

**Filtering conditions (±[X] Å/°)**	**0.3**	**0.5**	**0.7**
**# of merged datasets**	4	6	9
**Empirical formula**	$C_9H_8O_2$	$C_9H_8O_2$	$C_9H_8O_2$
**Formula weight**	148.15	148.15	148.15
**Wavelength / Å**	0.4859	0.4859	0.4859
**Temperature / K**	293	293	293
**Crystal system**	Monoclinic	Monoclinic	Monoclinic
**Space group**	$P2_1/n$	$P2_1/n$	$P2_1/n$
***a* / Å**	5.648(7)	5.605(5)	5.632(7)
***b* / Å**	18.38(3)	18.56(4)	18.41(2)
***c* / Å**	7.66(1)	7.625(9)	7.662(9)
***β* / °**	100.0(2)	100.0(1)	100.3(1)
**Volume**	783(2)	781(2)	782(2)
Z	4	4	4
**ρ_{calc}**	1.257	1.259	1.259
**μ / mm^{-1}**	0.039	0.044	0.039
***F*(000)**	312	312	312
**Total reflections**	1495	2254	3391
**Unique reflections**	437	445	475
**Mean $I/\sigma(I)$**	5.8	7.2	11.0
**R_{int}**	0.164	0.126	0.162
**R_{merge}**	0.103	0.136	0.260
**R_{meas}**	0.123	0.157	0.279
**R_{pim}**	0.065	0.074	0.098
**$CC_{1/2}$**	0.994	0.989	0.999
**Completeness θ_{max} (θ_{full}) / %**	91.8 (91.8)	93.5 (93.5)	99.8 (99.8)
**Multiplicity**	3.42	5.07	7.14
**d_{max} / Å**	1.2	1.2	1.2
**R_1 [I>=2$\sigma(I)$]**	0.0874	0.0728	0.0678
**wR_2 (all data)**	0.2513	0.2107	0.2019
**GooF on F^2**	1.159	0.825	0.837
**Largest peak/hole eÅ^{-3}**	0.14/-0.15	0.21/-0.15	0.17/-0.11

5.2 Assessment of X-ray-induced effects

Prior to the SR-FT-SSX photocrystallography experiments, control measurements were performed to assess whether exposure to the synchrotron X-ray beam could induce conversion of α-*trans*-cinnamic acid (α-TCA) to α-truxillic acid. Representative microcrystals from the same batch used for the subsequent SR-FT-SSX study were mounted on Mitegen™ MicroMounts and subjected to extended data collections. Two experiments were performed, using 20% and 50% of the available synchrotron beam intensity, respectively. In each case, 1750 ϕ-*scan* images were collected over a total rotation range of 350°.

To evaluate the effect of accumulated X-ray dose, each 350° dataset was manually divided during processing into six smaller rotation datasets that closely replicated the later SR-FT-SSX collection strategy: five 60° wedges (300 images each) and one 50° wedge (250 images). This approach generated six sequential datasets corresponding to progressively increasing X-ray exposure.

The resulting β-angle values, used as an indicator of conversion to α-truxillic acid, are shown in Figures S22 and S23. Negligible changes in β were observed throughout the extended data collections at both beam intensities, indicating that prolonged synchrotron X-ray exposure does not induce significant photoproduct formation under the experimental conditions employed. These control experiments therefore confirm that the structural changes observed during the SR-FT-SSX study arise from laser irradiation and not from X-ray-induced effects.

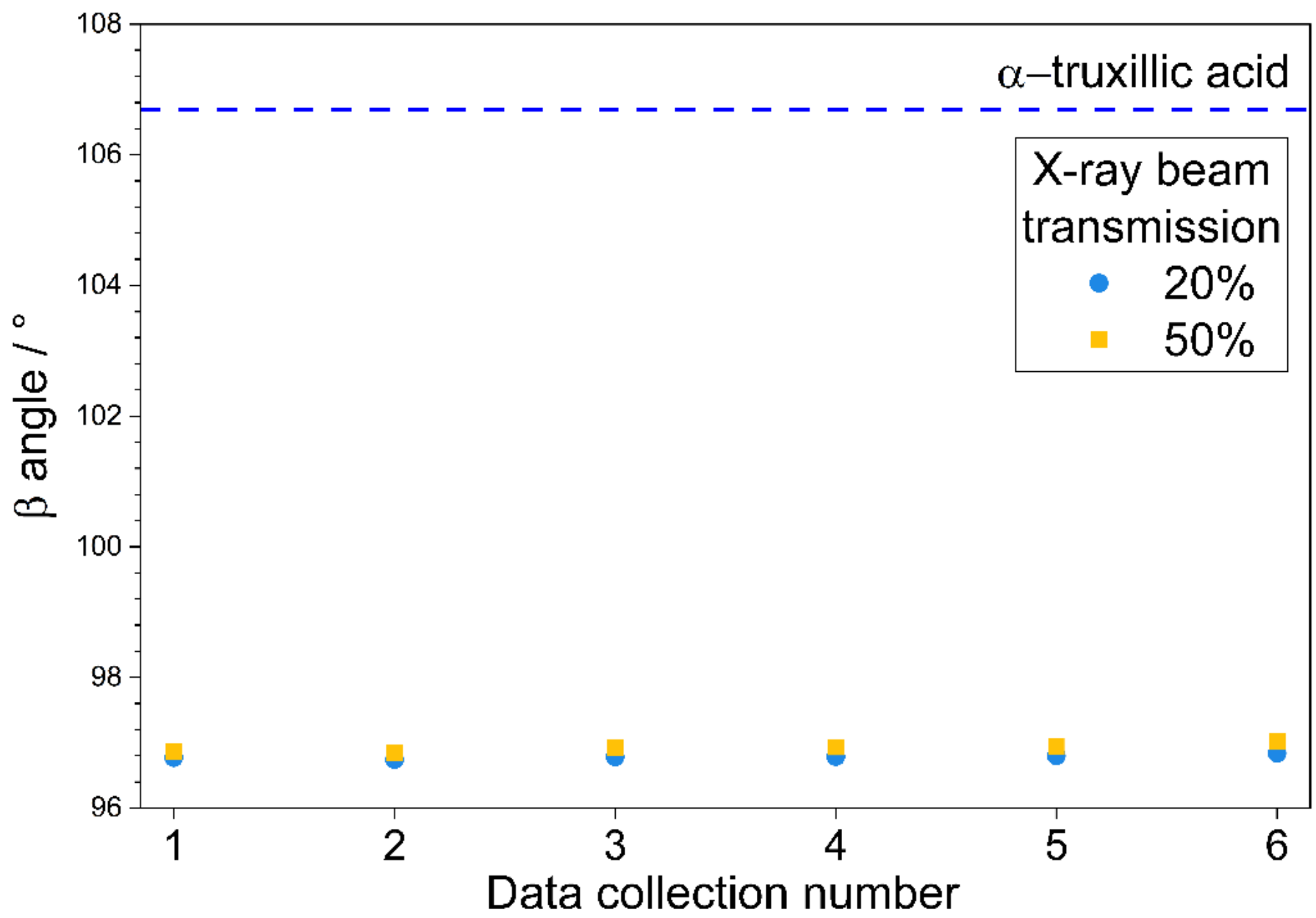

Figure S22 β-angle stability under prolonged X-ray exposure. The unit cell β angle is plotted as a function of cumulative X-ray exposure for datasets collected at 20% and 50% synchrotron beam transmission on Beamline I19, Diamond Light Source. X-ray dose increases with collection number. The dotted line indicates the reported β angle for fully converted α-truxillic acid.

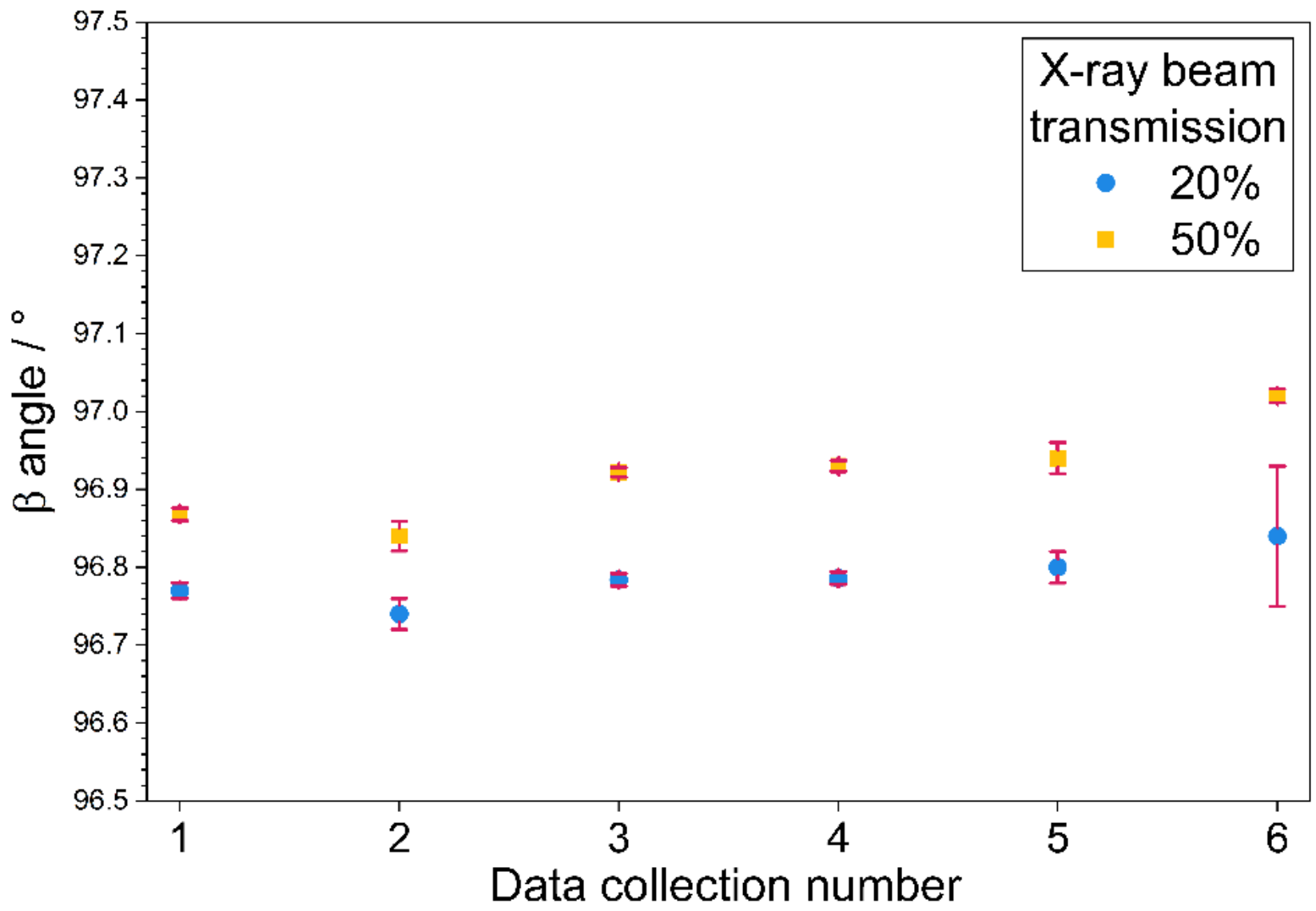

Figure S23. Enlarged view of β-angle variation with X-ray exposure. Expanded representation of the data shown in Figure S22, illustrating the change in unit cell β angle as a function of collection number (cumulative X-ray exposure). The enlarged scale allows the associated error bars to be displayed clearly.

Table S6 Unit cell parameters from X-ray exposure control experiments. Unit cell data obtained from sequential datasets collected during the prolonged X-ray exposure tests at 20% and 50% synchrotron beam transmission. Values are shown as a function of cumulative X-ray exposure to assess potential X-ray-induced changes in the crystal structure.

Data collection #	X-ray beam transmission / %	*a* (Å)	*b* (Å)	*c* (Å)	*α* (°)	*β* (°)	*γ* (°)
1	20	5.6299(6)	17.946(3)	7.7411(8)	90	96.77(1)	90
2	20	5.624(2)	17.915(2)	7.714(1)	90	96.74(2)	90
3	20	5.6422(4)	17.987(5)	7.7483(4)	90	96.784(8)	90
4	20	5.6417(4)	17.981(2)	7.7463(6)	90	96.786(8)	90
5	20	5.628(1)	17.955(2)	7.739(1)	90	96.80(2)	90
6	20	5.6398(5)	18.016(6)	7.7537(5)	90	96.84(9)	90
1	50	5.6302(5)	17.968(2)	7.7416(6)	90	96.868(8)	90
2	50	5.624(1)	17.945(2)	7.720(1)	90	96.84(2)	90
3	50	5.6381(3)	18.004(4)	7.7452(4)	90	96.922(6)	90
4	50	5.6304(4)	17.976(2)	7.7337(6)	90	96.930(7)	90
5	50	5.610(1)	17.949(2)	7.722(1)	90	96.94(2)	90
6	50	5.6388(5)	18.055(5)	7.7541(5)	90	97.020(9)	90

Section 6: Supplementary References

1. S. G. Lewis, B. A. Coulson, K. D. M. Harris, A. J. Warren, M. R. Warren and L. E. Hatcher, *CrystEngComm*, 2025, **27**, 4965 – 4975.
2. Southampton Nanofabrication Centre, University of Southampton <https://www.southampton.ac.uk/research/facilities/nanofabrication-cleanrooms> Accessed on 14/05/2026.
3. S. G. Lewis, B. A. Coulson, A. J. Warren, M. R. Warren and L. E. Hatcher, *Communications Chemistry*, 2024, **7**, 264.
4. J.-C. Bradley, M. H. Abraham, W. E. Acree, A. S. I. D. Lang, S. N. Beck, D. A. Bulger, E. A. Clark, L. N. Condron, S. T. Costa, E. M. Curtin, S. B. Kurtu, M. I. Mangir and M. J. McBride, *Chemistry Central Journal*, 2015, **9**, 11.
5. M. Aleksich, D. W. Paley, E. A. Schriber, W. Linthicum, V. Oklejas, D. W. Mittan-Moreau, R. P. Kelly, P. A. Kotei, A. Ghodsi, R. G. Sierra, A. Aquila, F. Poitevin, J. P. Blaschke, M. Vakili, C. J. Milne, F. Dall'Antonia, D. Khakhulin, F. Ardana-Lamas, F. Lima, J. Valerio, H. Han, T. Gallo, H. Yousef, O. Turkot, I. J. Bermudez Macias, T. Kluyver, P. Schmidt, L. Gelisio, A. R. Round, Y. Jiang, D. Vinci, Y. Uemura, M. Kloos, M. Hunter, A. P. Mancuso, B. D. Huey, L. R. Parent, N. K. Sauter, A. S. Brewster and J. N. Hohman, *Journal of the American Chemical Society*, 2023, **145**, 17042–17055.
6. C. A. Schneider, W. S. Rasband and K. W. Eliceiri, *Nature Methods*, 2012, **9**, 671–675.
7. S. D. M. Atkinson, M. J. Almond, P. Hollins and S. L. Jenkins, *Spectrochimica Acta Part A: Molecular and Biomolecular Spectroscopy*, 2003, **59**, 629–635.
8. S. E. Brown, M. R. Warren, D. J. Kubicki, A. Fitzpatrick and S. D. Pike, *Journal of the American Chemical Society*, 2024, **146**, 17325–17333.
9. P. Smyth, S. Jaho, L. J. Williams, G. Karras, A. Fitzpatrick, A. J. Thompson, S. Battah, D. Axford, S. Horrell, M. Lucic, K. Ishihara, M. Kataoka, H. Matsuura, K. Shimba, K. Tono, T. Tosha, H. Sugimoto, S. Owada, M. A. Hough, J. A. R. Worrall and R. L. Owen, *IUCrJ*, 2025, **12**, 582–594.
10. L. E. Hatcher, M. R. Warren, J. M. Skelton, A. R. Pallipurath, L. K. Saunders, D. R. Allan, P. Hathaway, G. Crevatin, D. Omar, B. H. Williams, B. A. Coulson, C. C. Wilson and P. R. Raithby, *Communications Chemistry*, 2022, **5**, 102.
11. G. Winter, D. G. Waterman, J. M. Parkhurst, A. S. Brewster, R. J. Gildea, M. Gerstel, L. Fuentes-Montero, M. Vollmar, T. Michels-Clark, I. D. Young, N. K. Sauter and G. Evans, *Acta Crystallographica Section D*, 2018, **74**, 85–97.
12. R. J. Gildea, D. G. Waterman, J. M. Parkhurst, D. Axford, G. Sutton, D. I. Stuart, N. K. Sauter, G. Evans and G. Winter, *Acta Crystallographica Section D*, 2014, **70**, 2652–2666.
13. J. B. Benedict and P. Coppens, *The Journal of Physical Chemistry A*, 2009, **113**, 3116–3120.
14. R. J. Gildea and G. Winter, *Acta Crystallographica Section D*, 2018, **74**, 405–410.
15. G. Sheldrick, *Acta Crystallographica Section A*, 2015, **71**, 3–8.
16. G. Sheldrick, *Acta Crystallographica Section C*, 2015, **71**, 3–8.
17. I. Abdelmoty, V. Buchholz, L. Di, C. Guo, K. Kowitz, V. Enkelmann, G. Wegner and B. M. Foxman, *Crystal Growth & Design*, 2005, **5**, 2210–2217.
